# A Novel Betabaculovirus Isolated from the Monocot Pest *Mocis latipes* (Lepidoptera: Noctuidae) and the Evolution of Multiple-Copy Genes

**DOI:** 10.3390/v10030134

**Published:** 2018-03-16

**Authors:** Daniel M. P. Ardisson-Araújo, Ana Maria Rodrigues da Silva, Fernando L. Melo, Ethiane Rozo dos Santos, Daniel R. Sosa-Gómez, Bergmann M. Ribeiro

**Affiliations:** 1 Laboratory of Insect Virology, Department of Biochemistry and Molecular Biology, Federal University of Santa Maria, Santa Maria RS 97105-900, Brazil; daniel_ardisson@yahoo.com.br (D.M.P.A.-A.); ethi_rozo@hotmail.com (E.R.d.S.); 2 Laboratory of Baculovirus, Cell Biology Department, University of Brasilia, Brasília DF 70910-900, Brazil; anmag2001@yahoo.com.br (A.M.R.d.S.); flucasmelo@gmail.com (F.L.M.); 3 Embrapa-Soja, Distrito de Warta P.O. Box 231, Londrina PR 86001-970, Brazil; daniel.sosa-gomez@embrapa.br

**Keywords:** baculovirus genome, betabaculovirus, Mocis latipes granulovirus, *enhancin*, *granulin*, *endonuclease*-like, genome expansion, evolution

## Abstract

In this report, we described the genome of a novel baculovirus isolated from the monocot insect pest *Mocis latipes*, the striped grass looper. The genome has 134,272 bp in length with a G + C content of 38.3%. Based on the concatenated sequence of the 38 baculovirus core genes, we found that the virus is a betabaculovirus closely related to the noctuid-infecting betabaculoviruses including Pseudaletia unipuncta granulovirus (PsunGV), Trichoplusia ni granulovirus (TnGV), Helicoverpa armigera granulovirus (HearGV), and Xestia c-nigrum granulovirus (XecnGV). The virus may constitute a new *Betabaculovirus* species tentatively named Mocis latipes granulovirus (MolaGV). After gene content analysis, five open reading frames (ORFs) were found to be unique to MolaGV and several auxiliary genes were found including *iap-3*, *iap-5*, *bro-a*, *bro-b*, and three *enhancins*. The virus genome lacked both *chitinase* and *cathepsin*. We then looked at the evolutionary history of the *enhancin* gene and found that betabaculovirus acquired this gene from an alphabaculovirus followed by several duplication events. Gene duplication also happened to an *endonuclease*-like gene. Genomic and gene content analyses revealed both a strict collinearity and gene expansion into the genome of the MolaGV-related species. We also characterized the granulin gene using a recombinant Autographa californica multiple nucleopolyhedrovirus (AcMNPV) and found that occlusion bodies were produced into the nucleus of infected cells and presented a polyhedral shape and no occluded virions within. Overall, betabaculovirus genome sequencing is of importance to the field as few genomes are publicly accessible. *Mocis* *latipes* is a secondary pest of maize, rice, and wheat crops in Brazil. Certainly, both the discovery and description of novel baculoviruses may lead to development of greener and safer pesticides in order to counteract and effectively control crop damage-causing insect populations

## 1. Introduction

The genus *Mocis* (Lepidoptera: Noctuidae) carries some important polyphagous insects that affect several cultures in many countries of the world [1]. These species are widespread throughout the American continent, impacting grasses, wheat, corn, rice, cotton, coffee, soybeans, and peanuts [2]. In Brazil, *Mocis* spp., especially *M. latipes* (Guenèe, 1852), also known as the striped grass looper, are pests of secondary importance. In some areas, high caterpillar infestations may occur, which demands immediate control to avoid economic losses in pastures. In this agro-ecosystems the use of a selective pesticide, such as baculovirus-based products, are particularly important to avoid toxic residues in the environment.

Baculoviruses are insect-specific viruses that cause a lethal disease in the larval stage of some lepidopteran, hymenopteran, and dipteran hosts. The family *Baculoviridae* is currently divided into four genera, *Alpha*-, *Beta*-, *Gamma*- and *Deltabaculovirus*. The International Committee on Taxonomy of Viruses (ICTV) taxonomy release in 2016 recognized 66 species in this family and only four did not present complete genome sequence publicly available. The GenBank database presents the complete sequence of almost two hundred genomes of baculoviruses with less than half kept as reference of unique species. Most of those sequences came from alphabaculovirus (47/73) and the lack of genomic information from the genera *Gamma*-, *Delta*- and *Betabaculovirus* hampers the evolutionary understanding of the *Baculoviridae* family.

Betabaculoviruses are infectious to lepidopteran hosts and present granular-shaped occlusion bodies (OBs) that protect the occluded virus from environmental adversities. The OB morphology is the reason the genus used to be called granuloviruses (GVs) [3] and the term granulovirus remains in the virus species name followed by the host species name.

In this work, we described the complete genome of a novel betabaculovirus isolated from an insect extract that was kept for several years in a freezer labeled as “Mocis sp. granulovirus” from the virus collection of The Brazilian Agricultural Research Corporation (Portuguese acronym EMBRAPA, Empresa Brasileira de Pesquisa Agropecuária). The relationship of this potentially novel virus to other baculovirus species and the evolution the *enhancin* and another multi-copy gene were analyzed. We also identified the species *Mocis latipes* as the host where the putative granulovirus was isolated and called this new virus, Mocis latipes granulovirus (MolaGV). Furthermore, we characterized the MolaGV *granulin* gene in a context of a recombinant alphabaculovirus infection and we found a very peculiar feature regarding its structure.

## 2. Materials and Methods

### 2.1. Virus Sample

Insect cadavers of some subjects from the genus *Mocis* sp. (not identified at species level until this work) were collected in the Southern Brazil (Colorado, Paraná state) in 1984, with symptoms of baculovirus infection. The cadavers were sent to EMBRAPA, and kept in freezer for further characterization. A small drop of the *Mocis*-labeled insect extract was subjected to light microscopy, and some small granular-shaped occlusion bodies (OBs) with Brownian motion were observed. Since granules are difficult to see by light microscopy we purified these OBs by centrifugation through a sucrose gradient as described elsewhere [4].

### 2.2. Viral Genomic DNA Extraction and Amplification

For host identification at the species level, 10 µL the virus containing-extract were centrifuged to separate the granules. We subjected the supernatant to phenol-chloroform DNA extraction as described elsewhere [5]. The purified DNA was used in a PCR reaction in order to amplify a lepidopteran *cytochrome oxidase I* (*coi*) gene fragment as previously described [6]. The amplified DNA was then sequenced and the sequence analyzed by BLASTN. Furthermore, 100 µL of the OB-containing suspension (10^5^ OBs/mL of ddH_2_O) were heated for 20 min at 95 °C, placed into ice for 5 min and treated with RQ1 RNase-Free DNase (Promega, Madison, WI, USA). The suspension was washed three times with SDS 0.5% and once with NaCl 0.5 M by centrifuging (7000× *g* for 10 min) and resuspending with equal volumes. The last resulting pellet was resuspended in ddH_2_O. The DNAse-treated OBs were dissolved in alkaline solution and used to extract DNA [4]. The DNA pellet was dissolved in 10 µL of sterile ddH_2_O at 50 °C for 1 h and directly subjected to a rolling circle amplification (RCA) reaction using the phi29 DNA polymerase and a random 3′ thiophosphate-protected hexamer primer according to the manufacturer’s protocols (New England Biolabs, Ipswich, MA, USA). Both quantity and quality of the purified DNA were determined by electrophoresis on a 0.8% agarose gel [5], visualized, and photographed in AlphaImager^®^ Mini (Alpha Innotech, San Leandro, CA, USA).

### 2.3. Genome Sequencing, Assembly, and Annotation

The viral genomic DNA was sequenced with the 454 Genome Sequencer (GS) Titanium at Macrogen Company (Seoul, South Korea). The genome was assembled de novo using Geneious 9.0 (Biomatters, Auckland, New Zealand) with a pairwise identity of 98.1% [7] into one single circular contig. The open reading frames (ORFs) that started with a methionine codon (ATG) and encoded polypeptides of at least 50 amino acids were identified with Geneious 9.0 and annotated using BLAST-X [8]. The genomic DNA sequence was submitted to GenBank under the accession number KR011718.

### 2.4. Phylogenetic Analyses and Genome Comparison

For *Baculoviridae* phylogenetic analysis, a Multiple Alignment using Fast Fourier Transform (MAFFT) alignment [9] was carried out with the concatenated nucleotide sequences of the 38 baculoviral core genes from several baculovirus genomes publicly available (Table S1). A maximum likelihood tree was inferred using the Fast-tree method [10] and a Shimodaira-Hasegawa-like test for branch support [11]. Moreover, the MolaGV complete genome was compared to other betabaculovirus genomes through construction of syntenic maps with the progressive Mauve algorithm implemented in the Geneious 9 with the default parameters. For the *enhancins* and *endonuclease-like* genes, a MAFFT alignment was carried out with the predicted amino acid sequence of the MolaGV genes and homologs. The hypothetical trees were inferred using the Randomized Axelerated Maximum Likelihood (RaxML) [12] under the model LG + I + G + F selected by Prottest 2.4 [13] and the Fast-tree methods, respectively. We collected the loci for all *enhancin*-containing betabaculovirus to check for duplication and synteny of the genes. Based on both, synteny and phylogeny we reconstructed the phylogenetic history of the *enhancins* into the betabaculovirus genus.

### 2.5. Gene Amplification, Shuttle Vectors, and Recombinant AcMNPV Virus Construction

The occlusion body main protein gene from MolaGV (*granulin*, MolaGV-ORF001) and Autographa californica multiple nucleopolyhedrovirus (AcMNPV) (*polyhedrin*, AcMNPV-ORF008) were separately amplified using two sets of primers (Polh-Ac-F TCCGAATTCATGCCGGATTATTCATACCGTCCC and Polh-Ac-R TCCCTGCAGTTAATACGCCGGACCAGTGAACAG; Gran-Mola-F TTCGAATTCATGGGATACAACAAATCATTGAGATAC and Gran-Mola-R TTCCTGCAGTTAGTAAGCGGGTCCGGTGAACAAAGG) in two reactions which contained 100 ng of the DNA-template (MolaGV or AcMNPV-C6 genomes), 300 µM of dNTP mix (Fermentas, Pittsburgh, PA, USA), 0.4 µM of each set of primer pairs, 1 U of VENT Polymerase (New England Biolabs, Ipswich, MA, USA), and 1× of the supplied reaction buffer. The reactions were subjected to the following program: 95 °C/2 min, 35 cycles of 95 °C/ 30 s, 55 °C/30 s and 68 °C/1 min with a final extension of 5 min at 68 °C. The amplified fragments were digested with *Eco*RI/*Pst*I (New England Biolabs, Ipswich, MA, USA) and cloned into pFastBac1^®^ (pFB1—Invitrogen, Carlsbad, CA, USA). The modified plasmids containing the heterologous genes were transformed into DH10-Bac cells (Invitrogen, Carlsbad, CA, USA) by heat shock, following the manufacturer’s instructions. Recombinant bacmids were selected and confirmed by PCR following the manufacturer’s instructions (Bac-to-Bac^®^, Baculovirus expression systems, Invitrogen, Carlsbad, CA, USA). One µg of each recombinant bacmid was transfected into 10^6^ insect IPLB-SF-21 (Sf21) cells [14] using Lipofectin following the manufacturer’s instructions (Invitrogen, Carlsbad, CA, USA). The supernatant of seven days post-transfection cells containing the recombinant viruses were collected, amplified in Sf21 cells, and titered as previously described [4].

### 2.6. Recombinant Protein Analysis by SDS-PAGE

For recombinant protein analysis Sf21 cells (3.5 × 10^6^ cells) were infected separately with the recombinant viruses (multiplicity of infection, MOI, of 5) and the infected cells were collected at 72 h post-infection (p.i.), washed three times with phosphate buffered saline (PBS) (pH 7.4) followed by steps of centrifugation (5000× *g*). The resulting pellets were resuspended in PBS, mixed with the same volume of loading buffer (0.25 M Tris-HCl, pH 6.8, 4% SDS, 20% glycerol, 10% 2-mercaptoethanol, and 0.02% bromophenol blue), heated for 5 min at 100 °C and subjected to electrophoresis in 12% SDS-PAGE gels, using the Mini Protean Tetra Cell apparatus (BioRad, Hercules, CA, USA) following the manufacturer’s instructions. The gels were then photographed using the ImageQuant™ LAS 4000, following the manufacturer’s instructions (GE, Boston, MA, USA).

### 2.7. Microscopy

For light microscopy, monolayers of Sf21 (5 × 10^6^) cells were infected separately with one of the two recombinant viruses at a MOI of 5. The infected cells were observed and photographed at different hours post-infection (h p.i.) in an Axiovert 100 inverted light microscope (Zeiss, Oberkochen, Germany). For transmission electron microscopy (TEM), Sf21 cells (5 × 10^6^) were infected as above and at 72 h p.i., the cells were fixed for 2 h in Karnovsky fixative (2% glutaraldehyde, 2% paraformaldehyde in 0.1 M sodium cacodylate buffer pH 7.4 with 5% sucrose), post-fixed (1% osmium tetroxide, 0.8% potassium ferricyanide in 0.1 M sodium cacodylate buffer pH 7.4), contrasted with 0.5% uranyl acetate, dehydrated in acetone, and embedded in Spurr’s resin. The ultrathin sections were obtained in an ultramicrotome (Leika ultracut UCT, Wetzlar, Germany), contrasted with uranyl acetate/lead citrate and observed in a TEM Jeol 1011 (Akishima, Tokyo, Japan) at 80 kV. For scanning electron microscopy (SEM) analysis of OBs, two infections were separately performed in Sf21 cells at 80% confluency in cell culture flasks (75 cm^2^) and with an MOI of 5. Cell monolayers were separately incubated for 1 h with the recombinant viruses, washed twice with TC-100 medium, and replenished with 12 mL fresh TC-100 medium supplemented with 10% Fetal Bovine Serum (FBS). The cells and OBs were collected at 120 h p.i. and OBs purified as described elsewhere [4]. The OBs suspensions (100 µL) were analyzed by scanning electron microscopy (SEM) according to previously published protocol [15].

## 3. Results and Discussion

### 3.1. Sample Evaluation and Genome Sequencing

The milky white purified putative occlusion bodies from the *Mocis*-labeled insect extract was shown by light microscopy to have small granular-shaped occlusion bodies (OBs) with Brownian motion, and were SDS resistant and alkaline sensitive. Indeed, all the features suggested that the sample contained a granulovirus. The same insect extract before virus purification was used for DNA extraction and identification of the host at the species level based on the mitochondrial *coi* gene [6]. The host was identified as belonging to the species *Mocis latipes* (Lepidoptera: Noctuidae).

The viral DNA was extracted and used for sequencing using the 454 Genome Sequencer (GS) FLX™ Titanium (Macrogen Inc., Seoul, Korea). Over 20,690 single-end reads were obtained after size and quality trimming (average size of 676.1 ± 207.8 nt with Q30 = 82.3%) and used for de novo assembling. We mapped 18,686 reads in one single circular contig of 134,272 bp long with a mean coverage 94.9 ± 30.9 times and a G + C content of 38.3%. We searched for Open Reading Frames (ORFs) starting with a methionine codon and at least 50 predicted amino acid residues in size and 145 ORFs, including all the currently defined baculovirus core genes, were found (Table S2). Six homologous repeat regions (hrs) ranging from 245 to 696 base pairs (bp) were found (Figure S1). Five hrs showed the consensus repeat “aaattttaatgtcgatct” and one hr showed a longer consensus repeat “atagcaggaatcaatttgtgcatggc” (Figure S1).

### 3.2. Virus Phylogeny

**Figure 1:**
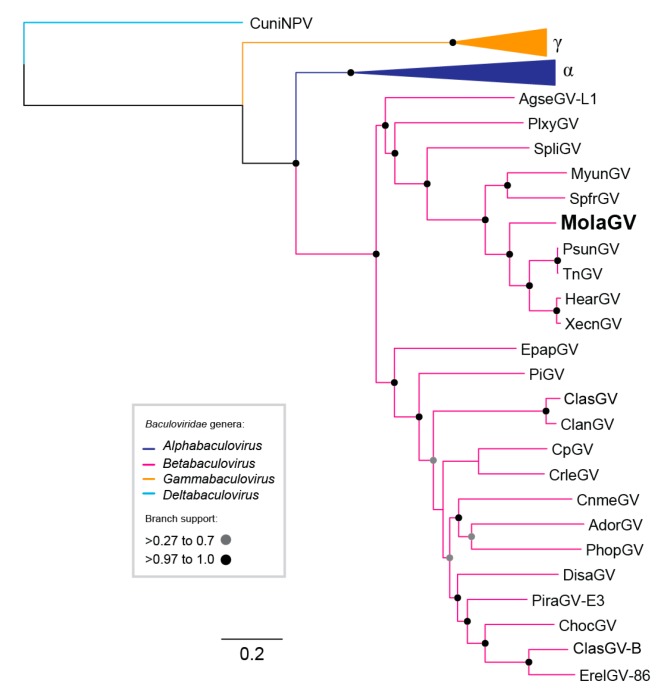
Maximum-likelihood tree for the *Baculoviridae*. The phylogenetic inference was based on the concatenated amino acid sequences of the 38 core proteins identified in all baculovirus genomes sequenced so far. We collapsed species from the genera *Gammabaculovirus* (orange, γ) and *Alphaphabaculovirus* (dark blue, α). Culex nigripalpus nucleopolyhedrovirus (CuniNPV) roots the tree (light blue). Mocis latipes granulovirus (MolaGV) (boldface) is a betabaculovirus and a sister species of the cluster formed by Pseudaletia unipuncta granulovirus (PsunGV), Trichoplusia ni granulovirus (TnGV), Xestia c-nigrum granulovirus (XecnGV), and Helicoverpa armigera granulovirus (HearGV) with high support.

Based on the alignment of the 38 baculovirus core proteins from several selected baculovirus genomes publicly available (Table S1), the virus was found to belong to the genus *Betabaculovirus* as a basal species of the clade formed by Pseudaletia unipuncta granulovirus (PsunGV), Trichoplusia ni granulovirus (TnGV), Xestia c-nigrum granulovirus (XecnGV), and Helicoverpa armigera granulovirus (HearGV) (Figure 1). This putative new species was tentatively named by Mocis latipes granulovirus (MolaGV). The pairwise nucleotide identity of MolaGV core genes with all completely sequenced betabaculovirus is presented in Table S1. Branch length separating the MolaGV from its closest relatives is in a range that is comparable to the branch lengths separating viruses in other recognized betabaculovirus species. A proposed baculovirus species demarcation criterion was published in 2006 that is based on pairwise nucleotide distances estimated using the Kimura 2-parameter model of nucleotide substitution for *lef-8*, *lef-9* and *polh/gran* genes [16]. Therefore, confirming MolaGV as a novel species, we found that the pairwise distances of sequences to other betabaculovirus are well in excess of 0.05 substitutions/site fulfilling the criteria for a novel betabaculovirus species (Table S3).

### 3.3. Gene Content

We searched for gene homologs using BLAST-X analysis. Only four ORFs (MolaGV-ORF026, MolaGV-ORF062, MolaGV-ORF094, and MolaGV-ORF140) were found to be unique with no predicted domains. Several auxiliary genes and a set of 19 betabaculovirus-specific genes [17] were also found in the genome of MolaGV. For instance, homologs of both *iap-3* (MolaGV-ORF108) and *iap-5* (MolaGV-ORF114) genes that are usually present in the genomes of betabaculoviruses were observed. These genes are involved in the anti-apoptotic response induced by virus infection [18]. The predicted *iap-3* (MolaGV-ORF108) homolog lacks one of the two conserved Baculovirus IAP Repeat (BIR) domains at the *N*-terminal region. This domain is thought to be involved in protein-protein interaction [19]. BLAST-X search revealed that the *iap-3* was closer to genes of alphabaculoviruses (Leucania separata nucleopolyhedrovirus (LeseNPV—46% identity, *e*-value = 5 × 10^−65^), Hyphantria cunea nucleopolyhedrovirus (HycuNPV—45% identity, *e*-value = 2 × 10^−63^) and lepidopteran than to genes of MolaGV-related granulovirus genes as expected. A phylogenetic reconstruction led us to believe that MolaGV underwent an independent acquisition of the *iap-3* during its evolution, similar to that observed for Plodia interpunctella granulovirus (PiGV) [20].

Only two *baculovirus repeated ORF* (*bro*) genes were found, *bro-a* (MolaGV-ORF058) and *bro-b* (MolaGV-ORF095) in the MolaGV genome. These genes belong to a unique multigenic family with unknown function [21]. Both the *bro-a* and *bro-b* genes are present in the Spodoptera frugiperda granulovirus (SpfrGV), PsunGV, HearGV, and XecnGV genomes and are clearly products of horizontal gene transfer (HGT) from alphabaculovirus. Moreover, the MolaGV genome lacks both *cathepsin* and *chitinase* genes which are implicated in virus horizontal transmission [22]. These genes are usually aside to each other in an opposite orientation in baculoviruses genomes [23]. Analyzing the available genomes of betabaculoviruses (22 genomes) we found that nine genomes have lost both genes, two have lost *cathepsin* and one has lost *chitinase* (Table S4).

### 3.4. Evolution of the Betabaculovirus Multiple Copy Genes: Enhancin and a Endonuclease-Like

**Figure 2:**
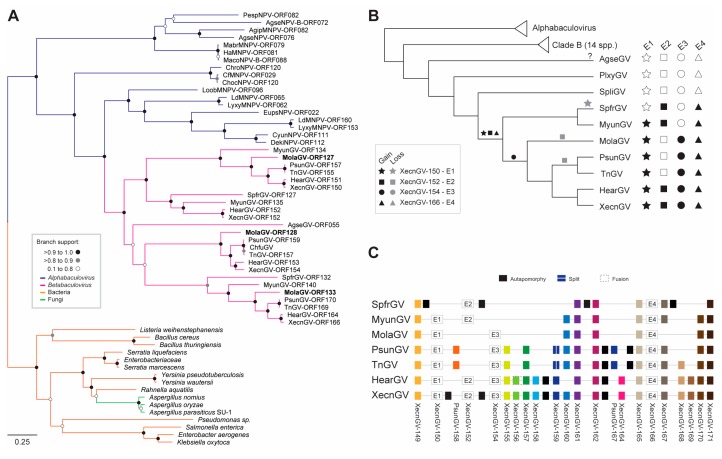
Enhancin evolution in MolaGV-related betabaculoviruses. (**A**) Phylogeny of *enhancin* homologs based on the predicted amino acid sequences by the Randomized Axelerated Maximum Likelihood (RaxML) method under LG + I + G + F model. We hypothesized that an alphabaculovirus (purple branches) ancestor acquired the gene once from bacteria (orange branches) as happened also to a specific group of fungus (green branches) and then the gene was transferred toward betabaculoviruses (pink branches). The tree was midpoint rooted and presented as a cladogram for clarity; (**B**) Acquisition and presence of four different forms of the *enhancin* genes of betabaculoviruses; (**C**) Genomic contexts of the *enhancin* genes confirm the ancestral acquisition of themselves. Rectangles with similar colors depict orthology, and the black rectangles independent acquisitions. We named the *enhancins* according to the appearance in the XecnGV genome annotation.

The MolaGV genome presents three *enhancins*. In a recent work describing the genome of the Trichoplusia ni granulovirus (TnGV), three *enhancin* genes were also observed [24]. The *enhancins* were described firstly in betabaculoviruses [25,26] and later found in some alphabaculoviruses (e.g., Choristoneura fumiferana multiple nucleopolyhedrovirus (CfMNPV), Choristoneura occidentalis nucleopolyhedrovirus (ChocNPV), Choristoneura rosaceana nucleopolyhedrovirus (ChroNPV), Dendrolimus kikuchii nucleopolyhedrovirus (DekiNPV), Lymantria dispar multiple nucleopolyhedrovirus (LdMNPV), Lymantria xylina nucleopolyhedrovirus (LyxyMNPV), Mamestra configurata nucleopolyhedrovirus B (MacoNPV-B), Agrotis segetum nucleopolyhedrovirus B (AgseNPV-B) [27,28]. In this work, we presented the evolutionary history of the *enhancins*, a multiple-copy gene (MolaGV-ORF126, MolaGV-ORF127, MolaGV-ORF132) (Figure 2). The phylogenetic reconstruction of the predicted amino acid sequence revealed that baculovirus *enhancin* genes were certainly acquired from bacteria (some Firmicutes species) (Figure 2A) and formed a monophyletic group, which suggests the occurrence of only one HGT event to baculoviruses. Two alternative hypotheses might be proposed to explain this observation: (i) the gene has been acquired by the ancestor of alpha- and betabaculoviruses and several independent losses explain its current distribution; or (ii) the gene has been firstly acquired from bacteria by some alphabaculovirus or betabaculovirus ancestor lineage and then, acquired by their counterpart. Indeed, the fact that the sequences from betabaculoviruses formed a monophyletic group within sequences from alphabaculoviruses (Figure 2A) supports the hypothesis that gene transfer occurred first to an alphabaculovirus lineage and after to betabaculoviruses rather than in the opposite direction.

Moreover, once introduced into the betabaculoviruses from an undisclosed alphabaculovirus-related source the gene underwent several duplications. This type of event seemed to happen also to the ancestors of LyxyMNPV and LdMNPV. In Figure 2B,C, we present the hypothesis of *enhancin* gene duplications undergone by betabaculoviruses. XecnGV presents four copies and we set them as reference and numbered each *enhancin* according to the ORF number annotated in the XecnGV genome (E1 to E4). The ancestor of all SpfrGV, MyunGV, MolaGV, PsunGV, TnGV, XecnGV, and HearGV probably acquired an *enhancing* gene once. This gene duplicated and gave rise to an E2-like gene (Figure 2B, square is *XecnGV-ORF152*) and an E4-like gene (Figure 2B, triangle is *XecnGV-ORF166*). The ancestor of MolaGV, PsunGV, TnGV, XecnGV, and HearGV underwent a double duplication event, by an E2-like gene that generated an E1-like gene (Figure 2B, star is *XecnGV-ORF150*) and by an E4-like gene that generated an E3-like gene (Figure 2B, circle is *XecnGV-ORF154*). Two independent losses of E2-like genes took place in both MolaGV and the ancestor of PsunGV and TnGV. Moreover, Agrotis segetum granulovirus (AgseGV) acquired independently an E4/E3-like gene. Indeed, all the four *enhancins* are syntenic and next to each other (Figure 2C) whereas the AgseGV gene is not, supporting the hypothesis of independent HGT from a granulovirus source. After whole genome alignment of three completely sequenced isolates of AgseGV, all genomes presented the *enhancin* gene at the same loci. However, we observed a variable amino-terminal region by MAFFT alignment. Auxiliary gene duplication was previously described in betabaculovirus evolution [15], despite the mechanism not being quite understood. Gene duplication is an important step for the emergence of new gene function and is crucial for evolution of enzymes, which are very sensitive to mutations that can lead to substrate biding activity changes.

Enhancins are known to be capable of enhancing baculoviruses (GVs and NPVs) infections in insect larvae [29]. Yang et al. [30] found that embedding the AgseGV-Enhancin protein into AcMNPV OBs improved the virus infectivity. There are two proposed models of action for Enhancin: (i) one is related to mucin degradation and permeability alteration of the insect midgut peritrophic membrane (PM) [31,32] and the other (ii) is related to their capacity to bind to larval midgut cells and probably serving as a binding protein for some viruses [33,34].

**Figure 3:**
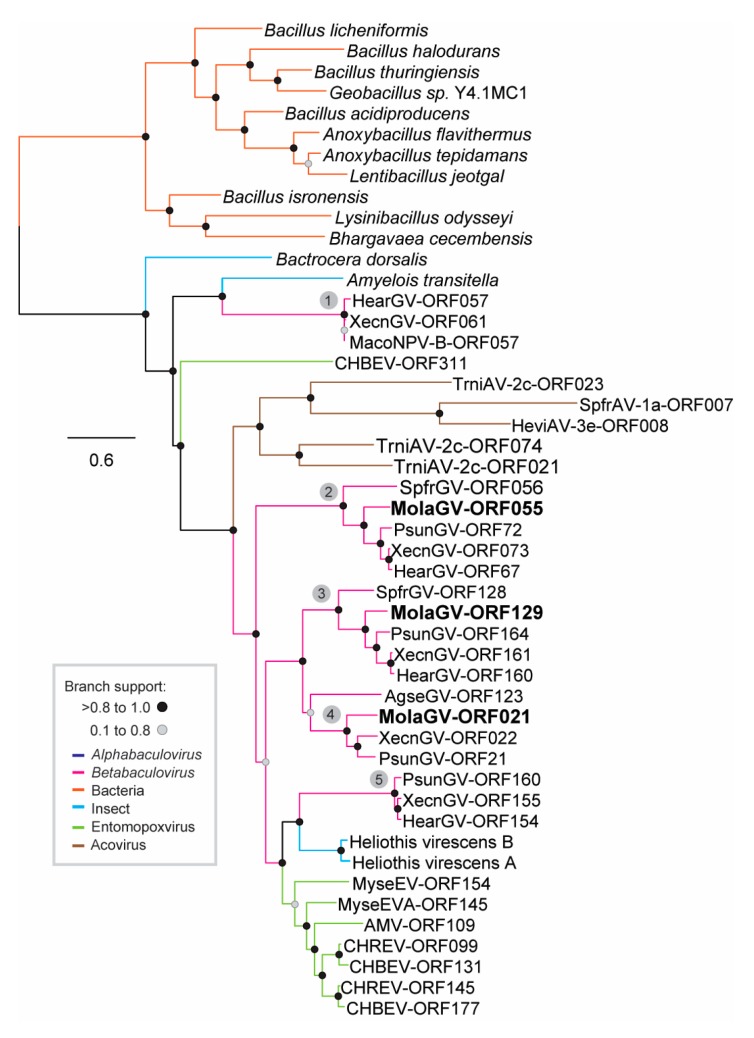
Recurrent duplication of an *endonuclease*-like gene in betabaculovirus. The phylogeny was based on the predicted amino acid sequence of Mola021, 055, and 129 homologs using maximum likelihood method implemented in FastTree. Betabaculovirus underwent one introduction from an undisclosed source that could be bacterial and several duplications took place in different betabaculovirus ancestors, creating five clusters numbered from 1 to 5. Cluster 5-related gene has been transferred to MacoNPV-B. Moreover, some ascovirus and entomopoxvirus genomes underwent a horizontal gene transfer (HGT) from betabaculovirus and the gene seemed to duplicate as well. The tree was midpoint rooted and presented as a cladogram for clarity.

Interestingly, the duplication is not restricted to the *enhancin* in the MolaGV genome. We found that the predicted amino acid sequence of MolaGV-ORF021, MolaGV-ORF055, and MolaGV-ORF129 presented a DNA/RNA non-specific endonuclease domain (NUC domain). By BLASTP search, MolaGV-ORF021, MolaGV-ORF055, and MolaGV-ORF129 had orthologs in MolaGV-related species and homologs in insects, entomopoxviruses, and ascoviruses. A signal peptide for secretion was found in most of the predicted proteins. The best Blast hits of these putative endonuclease sequences were with PsunGV with 69% amino acid identity for MolaGV-ORF021 (PsunGV-ORF21) and MolaGV-ORF055 (PsunGV-ORF72) and 70% for MolaGV129 (PsunGV-ORF164), with a coverage of 90%, 93% and 97%, respectively. All MolaGV putative endonucleases presented the DNA/RNA non-specific endonuclease (Pfam 13930:endonuclea_NS_2) domain at the C-terminal region. However, when comparing with a bacterial endonuclease with resolved structured (PDB: IQLO_A) the active sites were not conserved. As each of the genes had generated a very similar dataset during blast analyses, we asked whether they could potentially be products of duplication into the genome of betabaculoviruses. In an attempt to answer this question, we reconstructed the phylogeny of the genes. We found that betabaculovirus underwent one gene introduction from an undisclosed source, much likely from bacteria, and hence up to four gene duplications took place, creating five different gene clusters (Figure 3, numbered from 1 to 5). Alternatively, a horizontal gene transfer of the same genes could take place from another source. We also observed that betabaculovirus seemed to have transferred the gene to MacoNPV-B, entomopoxvirus (EV), and ascovirus (AV). A region into the MacoNPV-B genome is closely related and almost identical in some location to a cluster of XecnGV ORFs, suggesting recent recombination events between these two viruses [35]. Interestingly, once introduced into EV and AV genomes, the gene have duplicated as well. The function displayed by non-specific secreted endonuclease during insect virus infection is not clear. Only three insect groups were found to harbor a homolog of these endonucleases, *Bactrocera dorsalis* (Diptera: Tephritidae), *Amyelois transitella* (Lepidoptera: Pyralidae) and *Chloridea virescens* (Lepidoptera: Noctuidae). Both, the phylogeny and the branch support led us to hypothesize that those genes could be a HGT from viruses to insects. Moreover, *C. virescens* presents two alleles of this gene with an amino acid identity of 56%. Another endonuclease-expressing gene was found in the MolaGV genome, the MolaGV-ORF056 a homolog of *ac79*. This gene is a member of the UvrC superfamily of endonucleases involved in DNA repair [36].

### 3.5. Genome Expansion

**Figure 4:**
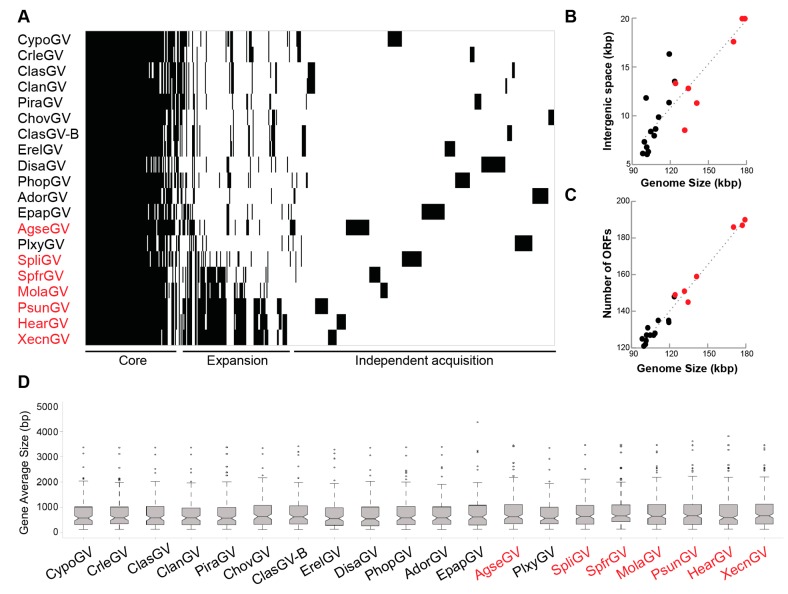
Genome expansion in the noctuid-infecting betabaculovirus lineage containing MolaGV. (**A**) A gene content matrix of selected betabaculoviruses. Black strips depict gene presence while white ones mean absence. Noctuid-infecting betabaculoviruses present a genome expansion (in red). The betabaculovirus core genes, the noctuid-infecting betabaculovirus-associated gene expansion and the independent acquisitions are indicated in the graph bottom; (**B**) Correlation between genome size and number of genes; (**C**) Correlation between genome size and intergenic spaces. Bigger the genome more intergenic spaces exist and more genes are potentially codified for; (**D**) The gene size is maintained across *Betabaculovirus*. Highlighted in red, noctuid-isolated betabaculovirus species. Most of these expanded open reading frames (ORFs) correspond to the betabaculovirus clade a specific ORFs identified in XecnGV clusters 1, 2, 3 and 4 described by [38].

Interestingly, MolaGV and the related species including *Spodoptera litura granulovirus* (SpliGV), SpfrGV, PsunGV, XecnGV, and HearGV present genome size much larger than betabaculoviruses average genome size (Table S1). However, the evolutionary force driving the size expansion it is not known. That could be related to the increase of the number of genes, their size, or the expansion of intergenic spaces. To answer this question and to investigate the reason for genome expansion, we analyzed the gene content of all completely sequenced genomes in the genus *Betabaculovirus* (Figure 4). A plot relating phylogeny and gene composition revealed three main set of genes (Figure 4A): a core set whereas all the genes are shared by the betabaculovirus species, an expansion set observed only for MolaGV-related species, and the set of unique genes. Interestingly, all the genomes harboring the related expansion are noctuid-infecting viruses (Figure 4, in red). When we looked at the correlation between genome size and both number of genes and sum of all intergenic regions in base pairs, we found a positive correlation (Figure 4B,C). Moreover, the average size of genes is maintained across the betabaculoviruses (Figure 4D). Therefore, the expansion happened by both events, gene and intergenic spaces acquisitions instead of the increase in gene size. However, the impact on virus fitness is unknown. Two important features are described for some of these species including XecnGV, HearGV, PsunGV, which are the slow speed of kill and the restricted tissue tropism [17]. Nevertheless, AgseGV seemed to have expanded independently in comparison to the other noctuid-infecting viruses by looking at their phylogeny. *Plutella xylostella granulovirus* (PlxyGV) is closely related to the MolaGV-related species when compared to AgseGV. Indeed, this betabaculovirus lineage containing most of the noctuid-infecting betabaculoviruses could tend to expand the genome in size when compared to the other lepidopteran family-isolated betabaculoviruses. This tendency must be investigated and must reflect the fluidity of baculovirus genomes as previously predicted [37].

### 3.6. Genomic Analysis

**Figure 5:**
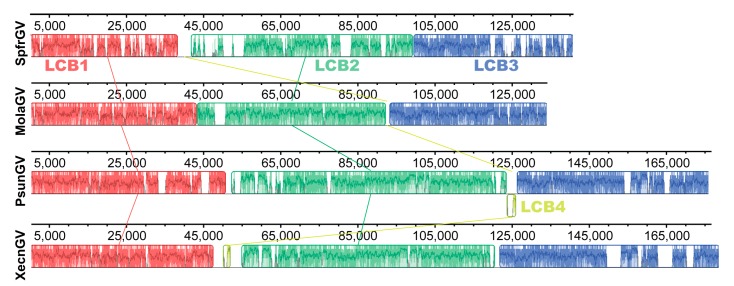
Genome comparison and gene content analyses of MolaGV and its related species. MolaGV sinteny is compared to three betabaculovirus genomes including SpfrGV, PsunGV, and XecnGV. Four Locally Collinear Blocks (LCB) numbered from 1 to 4 were found. As MolaGV presented smaller blocks in relation to both XecnGV and PsunGV, gene acquisition much probably took place on them. The main gain probably happened to the LCB2 region (green block) whereas LCB4 (yellow) is totally lacked by both the SpfrGV and MolaGV genome and seemed to undergo an inversion when XecnGV and PsunGV genomes are compared.

We performed a genomic comparison among some of the MolaGV-related species using progressive Mauve algorithm including SpfrGV, PsunGV, and XecnGV. Four Locally Collinear Blocks (LCB) were found and only three (LCB1, 2 and 3) were strictly conserved among these genomes (Figure 5). The regions are composed of genomic segments that appear to have the same relative position of their shared genes. Therefore, MolaGV and its relatives conserved a very strict genome collinearity despite of the difference in their genome sizes (Table S1). MolaGV is almost 40 kb smaller than its relatives leading us to believe that PsunGV, TnGV, HearGV, and XecnGV ancestors have suffered several gene gains during evolution. The acquisitions happened to the three LCBs (Figure 5). MolaGV relatives presented several genes lacked by MolaGV including *he65*-like, *cathepsin*, a few *bro* genes, *rep*-like, *chitinase*, *gp37*, *lef-7*, and numerous hypothetical genes. Moreover, LCB4 is not present in the MolaGV genome. This block harbors *bro* genes and some hypothetical ORFs including a homolog of an ascovirus-related *NDA-glutamate dehydrogenase* gene and an unknown entomopoxvirus-related gene. When comparing both XecnGV and PsunGV, there was a block inversion. New gene acquisitions may occur next to or on homologous regions, which usually present high content of *bro* genes as well. For instance, a recently described serine protease inhibitor found in the genome of Hemileuca sp. nucleopolyhedrovirus (HespNPV) was introduced into a hot spot for recombination with high content of repeat regions [38]. On the other hand, when we compared the MolaGV genome with its ancestor-related betabaculovirus species (i.e., SpfrGV), we found the opposite: a shortening of 6 kb on the genome size.

### 3.7. Characterization of the MolaGV Granulin Gene

**Figure 6:**
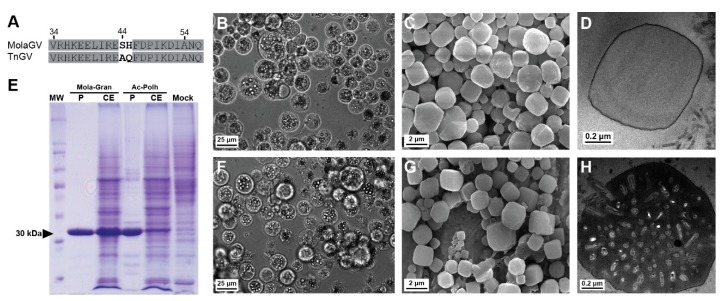
Characterization of the MolaGV *granulin* gene using a recombinant Autographa californica multiple nucleopolyhedrovirus (AcMNPV). (**A**) Specific region of the Granulin from both MolaGV and TnGV presenting the amino acid differences between the proteins; (**B**,**F**) SF-21 cells were infected for 72 h (Multiplicity of Infection, MOI, of 10) with either the MolaGV-*granulin*-expressing virus (**B**) or the AcMNPV-*polyhedrin*-expressing virus (**F**). The viruses produced crystals during infection and the crystals localized into the nuclei; (**C**,**G**) MolaGV-Granulin and AcMNPV-Polyhedrin crystals were similar in shape by SEM; (**D**) MolaGV-Granulin crystals were empty whereas (**H**) AcMNPV-Polyhedrin crystals presented embeded virions; (**E**) The crystals were also analyzed by SDS-PAGE and the head arrow points both the granulin and the polyhedrin band. Lane MW: protein marker; lane P: purified OBs: CE: virus-infected cell extracts. Mock: mock-infected cells.

In previous works, the substitution of the *granulin* gene from the Trichoplusia ni granulovirus (TnGV) for the *polyhedrin* of the AcMNPV and Bombyx mori nucleopolyhedrovirus (BmNPV) yielded a few very large (2 to 5 μm) cuboidal inclusions in the cytoplasm and nucleus of infected cells [39,40]. In a natural context, the *granulin* produces proteinaceous inclusions with a granular shape. TnGV belongs to the same clade of MolaGV betabaculovirus (Figure 1). When we compared the Granulin sequence from MolaGV and TnGV, we found two consecutive amino acid changes at the position 44 and 45 (Figure 6A). In an attempt to characterize the MolaGV *granulin* gene, we engineered the genome of AcMNPV, the type species of baculoviruses to express either its native *polyhedrin* gene from AcMNPV (vAc-AcMNPV-Polh) or the MolaGV *granulin* gene (vAc-MolaGV-Gran) under the *polh* gene promoter. In the natural context, both genes are able to produce crystalline inclusion bodies when highly expressed in infected cell during very late stages of virus infection progression. The MolaGV gene produces inclusion bodies with a granular shape whereas that from AcMNPV produces inclusion bodies with polyhedral shape. These crystals serve to protect the virion from environmental adversities [41]. In this work, during the recombinant virus infections, infected SF-21 cells produced crystals into the nuclei for both vAc-AcMNPV-Polh and vAc-MolaGV-Gran (Figure 6B,F) in a similar fashion. Moreover, the crystals resembled the native AcMNPV polyhedra in size and shape (Figure 6C,G) and not a cuboidal inclusion as observed for the TnGV granulin. We found also that the MolaGV-Gran-formed crystals were mostly empty (Figure 6D) whereas the AcMNPV-Polh-formed crystals presented multiple nucleocapsid virions within (Figure 6H), similar to that observed in a wild-type OB. To further confirm this result, we purified the crystals and subjected them to SDS-PAGE after alkaline solubilization (Figure 6E). We observed only one band when crystals formed by Mola-Granulin were resolved (Figure 6E, lane 2) and several faint bands together with a strongest band for crystals formed by AcMNPV-Polyhedrin (Figure 6E, lane 4). The crystal structure of baculoviruses’ OBs has been shown to be dependent of the amino acid sequence of the main occlusion body protein [42,43,44,45] and also on interactions with other viral and/or host proteins [46].

This is the first description of a polyhedral and not cuboidal crystals formed by a Granulin replacing the polyhedrin gene in AcMNPV. A single non-synonimous mutation in polyhedrin has been shown to be able to change drastically the crystal morphology and the ability to occlude virions [45,47,48]. We do not know why the expression of MolaGV *granulin* in Sf21 cells produced polyhedral-shaped OBs instead of the cuboidal-shaped OBs produced by recombinant BmNPV in Bm2 cells [39] and recombinant AcMNPV in *Trichoplusia ni* (BTI-TN-5B1-4) cells [40]. We can only speculate that the difference in shape could be due to the amino acid difference found in MolaGV when compared with TnGV sequence and/or the cells where the gene was expressed. Since we used a different cell line derived from *Spodoptera frugiperda* (Sf21), the shape of the protein could have been influenced by the interaction with specific cellular proteins. However, further studies will be necessary in order to find out what viral and/or host factors are necessary for the determination of baculoviruses OBs morphology.

## 4. Conclusions

In this work, we have described the genome of a baculovirus isolated from the noctuid *Mocis latipes*. The virus is a novel species into the genus *Betabaculovirus* that related itself as a basal group for the clade formed by the closest related species of XecnGV. It has shown to have 145 ORFs and only four were shown to be unique in the family *Baculoviridae*. Furthermore, several auxiliary genes were encountered. In particular, *enhancins* were found to undergo several duplications along betabaculovirus evolution. Moreover, we found that the betabaculovirus *enhancin* genes are a product of horizontal gene transfer from an alphabaculovirus. The duplication took place also in the *endonuclease*-like genes. MolaGV presented a very strict colinearity when compared to its relatives. Overall, betabaculovirus genome sequencing is of importance to the field as few genomes are publicly accessible. *Mocis latipes* is a secondary pest of pastures, wheat and maize crops in Brazil. Certainly, both discovery and description of novel baculoviruses may lead to the development of greener and safer pesticides in order to counteract and effectively control crop damage-causing insect population; moreover, that allow us to understand the evolution of baculovirus in a wider perspective.

## Supplementary Materials

The following are available online at http://www.mdpi.com/1999-4915/10/3/134/s1, Figure S1: MolaGV hrs, (A) Genome location of all hrs along the MolaGV genome. (B) Weblogo of the consensus repeat found in hr 1, 2, 3 5 and 6 (shown in purple). (C) Weblogo of the consensus repeat found in hr 4 (shown in blue), Table S1: Species used in this paper for the reconstruction of the baculovirus phylogeny in the Figure 1, Table S2: Characteristics of the Mocis latipes granulovirus (MolaGV) genome, Table S3: Evolutionary Divergence between MolaGV-related baculovirus using Kimura 2-parameter model of evolution, Table S4: Distribution of the chitinase and cathepsin in betabaculovirus genomes.

## References

[1] Assunção-Albuquerque M.J.T., Peso-Aguiar M.C., Albuquerque F.S. (2010). Using energy budget data to assess the most damaging life-stage of an agricultural pest *Mocis latipes* (Guenèe, 1982) (Lepidoptera-Noctuidae). Braz. J. Biol..

[2] Silvie P., Menzel C.A., Mello A., Coelho A.G. (2010). Population dynamics of caterpillars on three cover crops before sowing cotton in Mato Grosso (Brazil). Commun. Agric. Appl. Biol. Sci..

[3] Jehle J.A., Blissard G.W., Bonning B.C., Cory J.S., Herniou E.A., Rohrmann G.F., Theilmann D.A., Thiem S.M., Vlak J.M. (2006). On the classification and nomenclature of baculoviruses: A proposal for revision. Arch. Virol..

[4] O’Reilly D.R., Miller L.K., Luckow V.A. (1992). Baculovirus Expression Vectors: A Laboratory Manual.

[5] Sambrook J., Russell D.W. (2001). Molecular Cloning: A Laboratory Manual.

[6] Pinto F.A., Mattos M.V., Silva F.W., Rocha S.L., Elliot S.L. (2017). The Spread of Helicoverpa armigera (Lepidoptera: Noctuidae) and Coexistence with Helicoverpa zea in Southeastern Brazil. Insects.

[7] Kearse M., Moir R., Wilson A., Stones-Havas S., Cheung M., Sturrock S., Buxton S., Cooper A., Markowitz S., Duran C. (2012). Geneious Basic: An integrated and extendable desktop software platform for the organization and analysis of sequence data. Bioinformatics.

[8] Altschul S.F., Madden T.L., Schäffer A.A., Zhang J., Zhang Z., Miller W., Lipman D.J. (1997). Gapped BLAST and PSI-BLAST: A new generation of protein database search programs. Nucleic Acids Res..

[9] Katoh K., Misawa K., Kuma K.I., Miyata T. (2002). MAFFT: A novel method for rapid multiple sequence alignment based on fast Fourier transform. Nucleic Acids Res..

[10] Stamatakis A., Hoover P., Rougemont J. (2008). A rapid bootstrap algorithm for the RAxML web servers. Syst. Biol..

[11] Anisimova M., Gil M., Dufayard J.F., Dessimoz C., Gascuel O. (2011). Survey of branch support methods demonstrates accuracy, power, and robustness of fast likelihood-based approximation schemes. Syst. Biol..

[12] Guindon S., Dufayard J.F., Lefort V., Anisimova M., Hordijk W., Gascuel O. (2010). New algorithms and methods to estimate maximum-likelihood phylogenies: Assessing the performance of PhyML 3.0. Syst. Biol..

[13] Abascal F., Zardoya R., Posada D. (2005). ProtTest: Selection of best-fit models of protein evolution. Bioinformatics.

[14] Vaughn J.L., Goodwin R.H., Tompkins G.J., McCawley P. (1977). The establishment of two cell lines from the insect *Spodoptera frugiperda* (Lepidoptera; Noctuidae). Vitro.

[15] Ardisson-Araújo D.M.P., de Melo F.L., de Souza Andrade M., Sihler W., Báo S.N., Ribeiro B.M., de Souza M.L. (2014). Genome sequence of *Erinnyis ello granulovirus* (ErelGV), a natural cassava hornworm pesticide and the first sequenced sphingid-infecting betabaculovirus. BMC Genom..

[16] Jehle J.A., Lange M., Wang H., Hu Z., Wang Y., Hauschild R. (2006). Molecular identification and phylogenetic analysis of baculoviruses from Lepidoptera. Virology.

[17] Ferrelli M.L., Salvador R., Biedma M.E., Berretta M.F., Haase S., Sciocco-Cap A., Ghiringhelli P.D., Romanowski V. (2012). Genome of *Epinotia aporema granulovirus* (EpapGV), a polyorganotropic fast killing betabaculovirus with a novel thymidylate kinase gene. BMC Genom..

[18] Clem R.J. (2015). Viral IAPs, then and now. Semin. Cell Dev. Biol..

[19] Hinds M.G., Norton R.S., Vaux D.L., Day C.L. (1999). Solution structure of a baculoviral inhibitor of apoptosis (IAP) repeat. Nat. Struct. Biol..

[20] Harrison R.L., Rowley D.L., Funk C.J. (2016). The complete genome sequence of *Plodia interpunctella granulovirus*: Evidence for horizontal gene transfer and discovery of an unusual inhibitor-of-apoptosis gene. PLoS ONE.

[21] Bideshi D.K., Renault S., Stasiak K., Federici B.A., Bigot Y. (2003). Phylogenetic analysis and possible function of bro-like genes, a multigene family widespread among large double-stranded DNA viruses of invertebrates and bacteria. J. Gen. Virol..

[22] D’Amico V., Slavicek J., Podgwaite J.D., Webb R., Fuester R., Peiffer R.A. (2013). Deletion of v-chiA from a baculovirus reduces horizontal transmission in the field. Appl. Environ. Microbiol..

[23] Ishimwe E., Hodgson J.J., Clem R.J., Passarelli A.L. (2015). Reaching the melting point: Degradative enzymes and protease inhibitors involved in baculovirus infection and dissemination. Virology.

[24] Bivian-Hernández M.A., López-Tlacomulco J., Mares-Mares E., Ibarra J.E., Del Rincón-Castro M.C. (2017). Genomic analysis of a Trichoplusia ni Betabaculovirus (TnGV) with three different viral enhancing factors and two unique genes. Arch. Virol..

[25] Hashimoto Y., Corsaro B.G., Granados R.R. (1991). Location and nucleotide sequence of the gene encoding the viral enhancing factor of the *Trichoplusia ni granulosis* virus. J. Gen. Virol..

[26] Lepore L.S., Roelvink P.R., Granados R.R. (1996). Enhancin, the granulosis virus protein that facilitates nucleopolyhedrovirus (NPV) infections, is a metalloprotease. J. Invertebr. Pathol..

[27] Bischoff D.S., Slavicek J.M. (1997). Molecular analysis of an enhancin gene in the *Lymantria dispar nuclear polyhedrosis virus*. J. Virol..

[28] Popham H.J., Bischoff D.S., Slavicek J.M. (2001). Both *Lymantria dispar Nucleopolyhedrovirus* Enhancin Genes Contribute to Viral Potency. J. Virol..

[29] Guo H.F., Fang J.C., Wang J.P., Zhong W.F., Liu B.S. (2007). Interaction of *Xestia c-nigrum granulovirus* with peritrophic matrix and *Spodoptera litura nucleopolyhedrovirus* in *Spodoptera litura*. J. Econ. Entomol..

[30] Yang S., Zhao L., Ma R., Fang W., Hu J., Lei C., Sun X. (2017). Improving baculovirus infectivity by efficiently embedding enhancing factors into occlusion bodies. Appl. Environ. Microbiol..

[31] Peng J., Zhong J., Granados R.R. (1999). A baculovirus enhancin alters the permeability of a mucosal midgut peritrophic matrix from lepidopteran larvae. J. Insect Physiol..

[32] Wang P., Granados R.R. (1997). An intestinal mucin is the target substrate for a baculovirus enhancin. Proc. Natl. Acad. Sci. USA.

[33] Tanada Y. (1985). A synopsis of studies on the synergistic property of an insect baculoviurs: A tribute to Edward A. Steinhaus. J. Invertebr. Pathol..

[34] Hoover K., Humphries M.A., Gendron A.R., Slavicek J.M. (2010). Impact of viral enhancin genes on potency of *Lymantria dispar* multiple nucleopolyhedrovirus in *L. dispar* following disruption of the peritrophic matrix. J. Invertebr. Pathol..

[35] Li L., Donly C., Li Q., Willis L.G., Keddie B.A., Erlandson M.A., Theilmann D.A. (2002). Identification and genomic analysis of a second species of nucleopolyhedrovirus isolated from *Mamestra configurata*. Virology.

[36] Wu W., Passarelli A.L. (2012). The *Autographa californica* M nucleopolyhedrovirus ac79 gene encodes an early gene product with structural similarities to UvrC and intron-encoded endonucleases that is required for efficient budded virus production. J. Virol..

[37] Herniou E.A., Olszewski J.A., Cory J.S., O’Reilly D.R. (2003). The genome sequence and evolution of baculoviruses. Annu. Rev. Entomol..

[38] Harrison R.L., Rowley D.L., Mowery J., Bauchan G.R., Theilmann D.A., Rohrmann G.F., Erlandson M.A. (2017). The Complete Genome Sequence of a Second Distinct Betabaculovirus from the True Armyworm, *Mythimna unipuncta*. PLoS ONE.

[39] Ardisson-Araujo D.M., Rohrmann G.F., Ribeiro B.M., Clem R.J. (2015). Functional characterization of hesp018, a baculovirus-encoded serpin gene. J. Gen. Virol..

[40] Zhou C.E., Ko R., Maeda S. (1998). Polyhedron-like inclusion body formation by a mutant nucleopolyhedrovirus expressing the granulin gene from a granulovirus. Virology.

[41] Eason J.E., Hice R.H., Johnson J.J., Federici B.A. (1998). Effects of Substituting Granulin or a Granulin-Polyhedrin Chimera for Polyhedrin on Virion Occlusion and Polyhedral Morphology in Autographa californicaMultinucleocapsid Nuclear Polyhedrosis Virus. J. Virol..

[42] Rohrmann G.F. (2013). Baculovirus Molecular Biology.

[43] Carstens E.B., Williams G.V., Faulkner P., Partington S. (1992). Analysis of polyhedra morphology mutants of *Autographa californica* nuclear polyhedrosis virus: Molecular and ultrastructural features. J. Gen. Virol..

[44] Cheng X.W., Carner G.R., Fescemyer H.W. (1998). Polyhedrin sequence determines the tetrahedral shape of occlusion bodies in *Thysanoplusia orichalcea* single-nucleocapsid nucleopolyhedrovirus. J. Gen. Virol..

[45] Hu Z., Luijckx T., Van Dinten L.C., van Oers M.M., Haj J.P., Bianchi F.J., van Lent J.W., Zuidema D., Vlak J.M. (1999). Specificity of polyhedrin in the generation of baculovirus occlusion bodies. J. Gen. Virol..

[46] Ribeiro B.M., Generino A.P.M., Acacio C.N.L., Kalapothakis E., Báo S.N. (2009). Characterization of a new *Autographa californica multiple nucleopolyhedrovirus* (AcMNPV) polyhedra mutant. Virus Res..

[47] Woo S.D., Kim W.J., Kim H.S., Jin B.R., Lee Y.H., Kang S.K. (1998). The morphology of the polyhedra of a host range-expanded recombinant baculovirus and its parents. Arch. Virol..

[48] Ardisson-Araújo D.M.P., Rocha J.R., Da Costa M.H.O., Bocca A.L., Dusi A.N., de Oliveira Resende R., Ribeiro B.M. (2013). A baculovirus-mediated strategy for full-length plant virus coat protein expression and purification. Virol. J..

