# Supplementary material for: A Novel Betabaculovirus Isolated from the Monocot Pest Mocis latipes (Lepidoptera: Noctuidae) and the Evolution of Multiple-Copy Genes
Source: Viruses. 2018 Mar 16;10(3):134. doi: 10.3390/v10030134 (PMC5869527; doi:10.3390/v10030134)
Supplement: Supplementary file 1 [file viruses-10-00134-s001.zip › Table S1 (2).docx]

| **Table S1**. Species used in this paper for the reconstruction of the baculovirus phylogeny in the Fig. 1. Baculoviruses from the genera *Betabaculovirus* (pink), *Alphabaculovirus* (dark blue), *Gammabaculovirus* (orange), and *Deltabaculovirus* (light blue) are presented together with the acronym used in the main text, the host family where the virus was isolated from, the G+C content, the Genbank accession number, the genome size, and the global identity in relation to MolaGV (using a pairwise alignment of the concatenated nucleotide sequence of all the 38 baculovirus core genes). | | | | | | | |
| --- | --- | --- | --- | --- | --- | --- | --- |
| **Baculoviruses** | | **Acronym** | **Host family** | **G + C content (%)** | **Accession number** | **Genome Size (bp)** | **Id (%)** |
| 1 | Adoxophyes orana granulovirus | AdorGV | Tortricidae | 34.5 | AF547984 | 99657 | 49.0 |
| 2 | Agrotis segetum granulovirus isolate L1 | AgseGV-L1 | Noctuidae | 37.3 | KC994902 | 131442 | 51.0 |
| 3 | Choristoneura occidentalis granulovirus | ChocGV | Tortricidae | 32.7 | DQ333351 | 104710 | 50.1 |
| 4 | Clostera anastomosis granulovirus | ClasGV | Notodontidae | 46.7 | KC179784 | 101818 | 47.8 |
| 5 | Clostera anastomosis granulovirus B | ClasGV-B | [Notodontidae](https://en.wikipedia.org/wiki/Notodontidae) | 37.8 | KR091910 | 107409 | 49.4 |
| 6 | Clostera anachoreta granulovirus | ClanGV | Notodontidae | 44.4 | HQ116624 | 101487 | 48.0 |
| 7 | Cnaphalocrocis medinalis granulovirus | CnmeGV | [Crambidae](https://en.wikipedia.org/wiki/Crambidae) | 35.2 | KP658210 | 112060 | 46.8 |
| 8 | Cryptophlebia leucotreta granulovirus CV3 | CrleGV | Tortricidae | 32.4 | AY229987 | 110907 | 50.2 |
| 9 | Cydia pomonella granulovirus | CpGV | Tortricidae | 45.3 | U53466 | 123500 | 49.4 |
| 10 | Diatraea saccharalis granulovirus | DisaGV | Crambidae | 34.9 | KP296186 | 98392 | 49.5 |
| 11 | Epinotia aporema granulovirus | EpapGV | Tortricidae | 41.5 | JN408834 | 119082 | 49.5 |
| 12 | Erinnyis ello granulovirus | ErelGV | Sphingidae | 38.7 | KJ406702 | 102759 | 49.5 |
| 13 | Helicoverpa armigera granulovirus | HaGV | Noctuidae | 40.8 | EU255577 | 169794 | 74.9 |
| 14 | **Mocis latipes granulovírus** | **MolaGV** | **Noctuidae** | **38.3** | **KR011718** | **134272** | **100.0** |
| 15 | Mythimna unipuncta granulovírus | MyunGV#8 | Noctuidae | 49.9 | KX855660 | 144.673 | 66.0 |
| 16 | Phthorimaea operculella granulovirus | PhopGV | Gelechiidae | 35.7 | AF499596 | 119217 | 48.7 |
| 17 | Pieris rapae granulovirus isolate E3 | PiraGV-E3 | Pieridae | 33.2 | GU111736 | 108476 | 50.9 |
| 18 | Plodia interpunctella granulovirus | PiGV | [Pyralidae](https://pt.wikipedia.org/wiki/Pyralidae) | 44.2 | KX151395 | 112536 | 49.1 |
| 19 | Plutella xylostella granulovirus | PlxyGV | Plutellidae | 40.7 | AF270937 | 100999 | 49.3 |
| 20 | Pseudaletia unipuncta granulovirus | PsunGV-Hawaiin | Noctuidae | 39.8 | EU678671 | 176677 | 75.3 |
| 21 | Spodoptera frugiperda granulovirus | SpfrGV | Noctuidae | 46.2 | KM371112 | 140913 | 66.0 |
| 22 | Spodoptera litura granulovirus K1 | SpliGV | Noctuidae | 38.8 | DQ288858 | 124121 | 53.8 |
| 23 | Trichoplusia ni granulovirus | TnGV | Noctuidae | 39.8 | KU752557 | 175360 | 75.4 |
| 24 | Xestia c-nigrum granulovirus | XcGV | Noctuidae | 40.7 | AF162221 | 178733 | 74.4 |
| 25 | Adoxophyes honmai nucleopolyhedrovirus | AdhoNPV | Tortricidae | 35.6 | AP006270 | 113220 | 41.1 |
| 26 | Adoxophyes orana nucleopolyhedrovirus | AdorNPV | Tortricidae | 35.0 | EU591746 | 111724 | 41.3 |
| 27 | Agrotis ipsilon multiple nucleopolyhedrovirus strain illinois | AgipMNPV | Noctuidae | 48.6 | EU839994 | 155122 | 39.8 |
| 28 | Agrotis segetum nucleopolyhedrovirus | AgseNPV | Noctuidae | 45.7 | DQ123841 | 147544 | 40.2 |
| 29 | Antheraea pernyi nucleopolyhedrovirus isolate L2 | AnpeNPV-L2 | Saturniidae | 53.5 | EF207986 | 126246 | 37.5 |
| 30 | Anticarsia gemmatalis multiple nucleopolyhedrovirus | AgMNPV | Noctuidae | 44.5 | DQ813662 | 132239 | 38.6 |
| 31 | Apocheima cinerarium nucleopolyhedrovirus | ApciNPV | Geometridae | 33.4 | FJ914221 | 123876 | 37.7 |
| 32 | Autographa californica multiple nucleopolyhedrovirus C6 | AcMNPV-C6 | Noctuidae | 40.7 | L22858 | 133894 | 39.4 |
| 33 | Bombyx mandarina nucleopolyhedrovirus S2 | BomaNPV-S2 | Bombycidae | 40.4 | JQ071499 | 129646 | 39.3 |
| 34 | Bombyx mori nucleopolyhedrovirus strain T3 | BmNPV-T3 | Bombycidae | 40.4 | L33180 | 128413 | 39.4 |
| 35 | Buzura suppressaria nucleopolyhedrovirus | BusuNPV | Geometridae | 36.8 | KF611977 | 120420 | 40.1 |
| 36 | Catopsilia pomona nucleopolyhedrovirus | CapoNPV | Pieridae | 39.7 | KU565883 | 128058 | 38.8 |
| 37 | Choristoneura fumiferana defective multiple nucleopolyhedrovirus | CfDEFMNPV | Tortricidae | 45.8 | AY327402 | 131160 | 38.4 |
| 38 | Choristoneura fumiferana multiple nucleopolyhedrovirus | CfMNPV | Tortricidae | 50.1 | AF512031 | 129593 | 37.9 |
| 39 | Choristoneura murinana nucleopolyhedrovirus | ChmuNPV | Tortricidae | 50.0 | KF894742 | 124688 | 38.1 |
| 40 | Choristoneura occidentalis nucleopolyhedrovirus | ChocNPV | Tortricidae | 50.1 | KC961303 | 128446 | 37.9 |
| 41 | Choristoneura rosaceana nucleopolyhedrovirus | ChroNPV | Tortricidae | 48.6 | KC961304 | 129052 | 38.1 |
| 42 | Chrysodeixis chalcites nucleopolyhedrovirus | ChchNPV | Noctuidae | 39.0 | AY864330 | 149622 | 40.8 |
| 43 | Chrysodeixis includens single nucleopolyhedrovirus IF | ChinSNPV | Noctuidae | 39.2 | KU669293 | 139181 | 40.3 |
| 44 | Clanis bilineata nucleopolyhedrovirus | ClbiNPV | Sphingidae | 37.7 | DQ504428 | 135454 | 40.0 |
| 45 | Condylorrhiza vestigialis multiple nucleopolyhedrovirus | CoveMNPV | Crambidae | 42.9 | KJ631623 | 125767 | 38.7 |
| 46 | Cyclophragma undans nucleopolyhedrovirus | CyunNPV | Lasiocampidae | 45.1 | KT957089 | 140418 | 38.3 |
| 47 | Dasychra pudibunda nucleopolyhedrovirus | DapuNPV | Lymantriidae | 54.4 | KP747440 | 136761 | 37.6 |
| 48 | Dendrolimus kikuchii nucleopolyhedrovirus | DekiNPV | Lasiocampidae | 48.0 | JX193905 | 141454 | 38.1 |
| 49 | Ectropis obliqua nucleopolyhedrovirus strain A1 | EcobNPV-A1 | Geometridae | 37.6 | DQ837165 | 131204 | 40.4 |
| 50 | Epiphyas postvittana nucleopolyhedrovirus | EppoNPV | Tortricidae | 40.7 | AY043265 | 118584 | 38.7 |
| 51 | Euproctis pseudoconspersa nucleopolyhedrovirus | EupsNPV | Lymantriidae | 40.3 | FJ227128 | 141291 | 39.7 |
| 52 | Helicoverpa armigera multiple nucleopolyhedrovirus | HaMNPV | Noctuidae | 40.1 | EU730893 | 154196 | 40.8 |
| 53 | Helicoverpa armigera nucleopolyhedrovirus G4 | HaNPV-G4 | Noctuidae | 39.0 | AF271059 | 130759 | 40.4 |
| 54 | Helicoverpa zea single nucleopolyhedrovirus USA | HzSNPV-USA | Noctuidae | 39.1 | AF334030 | 130869 | 40.7 |
| 55 | Hemileuca sp. Nucleopolyhedrovirus | HespNPV | Saturniidae | 38.1 | KF158713 | 140633 | 38.8 |
| 56 | Hyphantria cunea nucleopolyhedrovirus | HycuNPV | Arctiidae | 45.5 | AP009046 | 132959 | 38.2 |
| 57 | Lambdina fiscellaria nucleopolyhedrovirus | LafiNPV | Geometriidae | 43.7 | KP752043 | 157977 | 37.9 |
| 58 | Leucania separata nuclear polyhedrovirus strain AH1 | LeseNPV | Noctuidae | 48.6 | AY394490 | 168041 | 39.7 |
| 59 | Lonomia obliqua multiple nucleopolyhedrovirus | LoobMNPV | Saturniidae | 35.7 | KP763670 | 120022 | 39.5 |
| 60 | Lymantria díspar multiple nucleopolyhedrovirus | LdMNPV | Lymantriidae | 57.5 | AF081810 | 161046 | 38.4 |
| 61 | Lymantria xylina multiple nucleopolyhedrovirus | LyxyMNPV | Lymantriidae | 53.5 | GQ202541 | 156344 | 39.1 |
| 62 | Mamestra brassicae multiple nucleopolyhedrovirus strain Chb1 | MbMNPV-CHb1 | Noctuidae | 40.1 | JX138237 | 154451 | 37.8 |
| 63 | Mamestra configurata nucleopolyhedrovirus B | MacoNPV-B | Noctuidae | 40.0 | AY126275 | 158482 | 40.8 |
| 64 | Mamestra configurata nucleopolyhedrovirus-A strain 90/2 | MacoNPV-A 90/2 | Noctuidae | 41.7 | U59461 | 155060 | 40.5 |
| 65 | Maruca vitrata multiple nucleopolyhedrovirus | MaviMNPV | Crambidae | 38.6 | EF125867 | 111953 | 39.6 |
| 66 | Operophtera brumata nucleopolyhedrovirus | OpbuNPV | Geometridae | 38.9 | - | 119054 | 40.8 |
| 67 | Orgyia leucostigma nucleopolyhedrovirus isolate CFS-77 | OrleNPV | Lymantriidae | 39.9 | EU309041 | 156179 | 40.2 |
| 68 | Orgyia pseudotsugata multiple nucleopolyhedrovirus | OpMNPV | Lymantriidae | 55.1 | U75930 | 131995 | 37.5 |
| 69 | Peridroma sp. nucleopolyhedrovirus | PespNPV | Noctuidae | 53.2 | KM009991 | 151109 | 39.6 |
| 70 | Perigonia lusca single nucleopolyhedrovirus | PeluSNPV | Sphingidae | 39.6 | KM596836 | 132831 | 40.6 |
| 71 | Philosamia cynthia ricini nucleopolyhedrovirus | PhcyNPV | Saturniidae | 53.7 | JX404026 | 125376 | 37.3 |
| 72 | Plutella xylostella multiple nucleopolyhedrovirus isolate CL3 | PlxyMNPV | Plutellidae | 40.7 | DQ457003 | 134417 | 39.4 |
| 73 | Rachiplusia ou multiple nucleopolyhedrovirus | RoMNPV | Noctuidae | 39.1 | AY145471 | 131526 | 39.4 |
| 74 | Spodoptera exigua nucleopolyhedrovirus | SeMNPV | Noctuidae | 43.8 | AF169823 | 135611 | 40.1 |
| 75 | Spodoptera frugiperda multiple nucleopolyhedrovirus isolate 19 | SfMNPV-I19 | Noctuidae | 40.3 | EU258200 | 132565 | 40.9 |
| 76 | Spodoptera litoralis nucleopolyhedrovirus isolate AN1956 | SpliNPV-1956 | Noctuidae | 44.7 | JX454574 | 137998 | 39.9 |
| 77 | Spodoptera litura nucleopolyhedrovirus G2 | SlNPV-G2 | Noctuidae | 42.8 | AF325155 | 139342 | 40.1 |
| 78 | Spodoptera litura nucleopolyhedrovirus II | SlNPV-II | Noctuidae | 45.0 | EU780426 | 148634 | 39.9 |
| 79 | Sucra jujuba nucleopolyhedrovirus | SujuNPV | Geometridae | 38.7 | KJ676450 | 135952 | 40.0 |
| 80 | Thysanoplusia orichalcea nucleopolyhedrovirus | ThorNPV | Noctuidae | 39.5 | JX467702 | 132978 | 39.6 |
| 81 | Trichoplusia ni single nucleopolyhedrovirus | TnSNPV | Noctuidae | 39.0 | DQ017380 | 134394 | 40.7 |
| 82 | Urbanus proteus nucleopolyhedrovirus | UrprNPV | Hesperiidae | 34.7 | KR011717 | 105555 | 40.6 |
| 83 | Neodiprion sertifer nucleopolyhedrovirus | NeseNPV | Diprionidae | 33.8 | AY430810 | 86462 | 34.4 |
| 84 | Neodiprion lecontei nucleopolyhedrovirus | NeleNPV | Diprionidae | 33.4 | AY349019 | 81755 | 34.3 |
| 85 | Neodiprion abietis nucleopolyhedrovirus | NeabNPV | Diprionidae | 33.4 | DQ317692 | 84264 | 34.1 |
| 86 | Culex nigripalpus nucleopolyhedrovirus | CuniNPV | Culicidae | 50.9 | AF403738 | 108252 | 28.5 |
