# Supplementary material for: A Novel Betabaculovirus Isolated from the Monocot Pest Mocis latipes (Lepidoptera: Noctuidae) and the Evolution of Multiple-Copy Genes
Source: Viruses. 2018 Mar 16;10(3):134. doi: 10.3390/v10030134 (PMC5869527; doi:10.3390/v10030134)
Supplement: Supplementary file 1 [file viruses-10-00134-s001.zip › Table S2 (2).docx]

**Table S2**. Characteristics of the Mocis latipes granulovirus (MolaGV) genome: number, position, nucleotide, and amino acid size of each ORF and homology search. Predicted ORFs are compared with homolog genes in two related genomes: Pseudaletia unipuncta granulovirus-Hawaiin (PsunGV-Hawaiin), Spodoptera frugiperda granulovirus-vg008 (SpfrGV-vg008), Xestia c-nigrum granulovirus (XecnGV), and the type species of granulovirus*,* Cydia pomonella granulovirus (CpGV).

|  |  |  |  | |  | |  | |  | **PsunGV-Hawaiin** | | **SpfrGV-vg008** | | | **XecnGV** | | | **CpGV** | | |
| --- | --- | --- | --- | --- | --- | --- | --- | --- | --- | --- | --- | --- | --- | --- | --- | --- | --- | --- | --- | --- |
| **ORF** | **Name** | **Position** | | | | **Size (nt)** | | **Size (aa)** | | **ORF** | **ID (%)** | **ORF** | **ID (%)** | **ORF** | | **ID (%)** | **ORF** | | **ID (%)** |  |
| 1 | *granulin* | 1 | > | 747 | | 747 | | 248 | | 1 | 99 | 1 | 98 | 1 | | 99 | 1 | | 87 |  |
| 2 | *orf1629* | 809 | < | 1.537 | | 729 | | 242 | | 2 | 51 | 2 | 69 | 2 | | 51 | 2 | | 15 |  |
| 3 | *pk-1* | 1.518 | > | 2.390 | | 873 | | 290 | | 3 | 79 | 3 | 67 | 3 | | 79 | 3 | | 44 |  |
| 4 | *MolaGV-ORF-4* | 2.421 | < | 3.260 | | 840 | | 279 | | 4 | 46 | 4 | 30 | 4 | | 40 | - | | - |  |
| 5 | *p10* | 3.423 | > | 3.677 | | 255 | | 84 | | 5 | 92 | 5 | 74 | 5 | | 89 | - | | - |  |
| 6 | *MolaGV-ORF-6* | 3.724 | < | 4.290 | | 567 | | 188 | | 6 | 69 | 7 | 56 | 7 | | 71 | 4 | | 32 |  |
| 7 | *cp5-like* | 4.280 | > | 4.546 | | 267 | | 88 | | 7 | 65 | 6 | 49 | 8 | | 63 | 5 | | 22 |  |
| 8 | *ie-1* | 4.605 | < | 6.026 | | 1.422 | | 473 | | 8 | 71 | 8 | 55 | 9 | | 64 | 7 | | 33 |  |
| 9 | *cp8-like* | 6.046 | > | 6.603 | | 558 | | 185 | | 9 | 75 | 9 | 54 | 10 | | 75 | 8 | | 33 |  |
| 10 | *cbm14-like domain* | 6.671 | < | 6.970 | | 300 | | 99 | | 10 | 93 | 10 | 80 | 11 | | 91 | 9 | | 53 |  |
| 11 | *odv-e18* | 6.991 | < | 7.239 | | 249 | | 82 | | 11 | 79 | 11 | 80 | 12 | | 79 | 14 | | 60 |  |
| 12 | *p49* | 7.248 | < | 8.606 | | 1.359 | | 452 | | 12 | 76 | 12 | 68 | 13 | | 75 | 15 | | 42 |  |
| 13 | *ring-domain* | 8.675 | < | 9.364 | | 690 | | 229 | | 13 | 77 | 13 | 61 | 14 | | 78 | - | | - |  |
| 14 | *odv-e56 (pif-5)* | 9.379 | < | 10.440 | | 1.062 | | 353 | | 14 | 77 | 14 | 70 | 15 | | 77 | 18 | | 57 |  |
| 15 | *MolaGV-ORF-15* | 10.477 | > | 10.686 | | 210 | | 69 | | 15 | 66 | 15 | 53 | 16 | | 55 | - | | - |  |
| 16 | *pep-1* | 10.716 | < | 11.282 | | 567 | | 188 | | 16 | 91 | 16 | 66 | 17 | | 87 | 20 | | 42 |  |
| 17 | *pep-2* | 11.328 | < | 11.792 | | 465 | | 154 | | 17 | 88 | 17 | 81 | 18 | | 91 | 23 | | 52 |  |
| 18 | *pep/p10* | 11.816 | < | 12.973 | | 1.158 | | 385 | | 18 | 88 | 18 | 76 | 19 | | 87 | 22 | | 48 |  |
| 19 | *cbm14-like domain* | 13.075 | > | 13.383 | | 309 | | 102 | | 19 | 47 | 134 | 37 | 20 | | 46 | - | | - |  |
| 20 | *p94* | 13.463 | > | 15.871 | | 2.409 | | 802 | | 20 | 63 | 132 | 11 | 21 | | 57 | - | | - |  |
| 21 | *MolaGV-ORF-21* | 15.918 | < | 17.426 | | 1.509 | | 502 | | 21 | 66 | 128 | 42 | 22 | | 62 | - | | - |  |
| 22 | *MolaGV-ORF-22* | 17.575 | < | 18.348 | | 774 | | 257 | | 22 | 66 | 19 | 57 | 23 | | 35 | - | | - |  |
| 23 | *MolaGV-ORF-23* | 19.468 | > | 20.721 | | 1.254 | | 417 | | 24 | 57 | 21 | 46 | 25 | | 54 | - | | - |  |
| 24 | *cp30-like* | 21.231 | > | 22.211 | | 981 | | 326 | | 25 | 47 | 22 | 27 | 26 | | 57 | 30 | | 10 |  |
| 25 | *efp* | 22.286 | > | 24.037 | | 1.752 | | 583 | | 26 | 71 | 23 | 55 | 27 | | 69 | 31 | | 33 |  |
| 26* | *MolaGV-ORF-26* | 23.979 | > | 24.182 | | 204 | | 67 | | - | - | - | - | - | | - | - | | - |  |
| 27 | *MolaGV-ORF-27* | 24.161 | > | 24.994 | | 834 | | 277 | | - | - | 24 | 33 | 28 | | 56 | - | | - |  |
| 28 | *cp33-like* | 24.987 | < | 25.685 | | 699 | | 232 | | 28 | 68 | 25 | 55 | 29 | | 69 | 33 | | 28 |  |
| 29 | *pif-3* | 25.746 | > | 26.336 | | 591 | | 196 | | 30 | 80 | 27 | 63 | 32 | | 79 | 35 | | 49 |  |
| 30 | *MolaGV-ORF-30* | 26.350 | > | 26.616 | | 267 | | 88 | | 31 | 71 | 28 | 46 | 33 | | 75 | - | | - |  |
| 31 | *cp39-like* | 26.626 | > | 26.976 | | 351 | | 116 | | 32 | 91 | 29 | 79 | 34 | | 90 | 39 | | 47 |  |
| 32 | *lef-2* | 26.978 | > | 27.544 | | 567 | | 188 | | 33 | 73 | 30 | 59 | 35 | | 78 | 41 | | 42 |  |
| 33 | *MolaGV-ORF-33* | 27.505 | > | 27.807 | | 303 | | 100 | | 34 | 68 | 31 | 29 | 36 | | 69 | - | | - |  |
| 34 | *MolaGV-ORF-34* | 27.850 | < | 28.305 | | 456 | | 151 | | 37 | 58 | 33 | 45 | 39 | | 55 | - | | - |  |
| 35 | *metalloproteinase* | 28.384 | < | 30.126 | | 1.743 | | 580 | | 38 | 57 | 34 | 48 | 40 | | 62 | 46 | | 31 |  |
| 36 | *MolaGV-ORF-36* | 30.139 | < | 31.377 | | 1.239 | | 412 | | 40 | 45 | 35 | 26 | 42 | | 50 | - | | - |  |
| - | *direct repeat 1* | 31.438 | > | 31.488 | | - | | - | | - | - | - | - | - | | - | - | | - |  |
| 37 | *p13* | 31.493 | > | 32.311 | | 819 | | 272 | | 41 | 76 | 36 | 71 | 43 | | 77 | 47 | | 51 |  |
| 38 | *MolaGV-ORF-39* | 32.562 | > | 33.041 | | 480 | | 159 | | 42 | 44 | - | - | 44 | | 39 | - | | - |  |
| 39 | *pif-2* | 33.048 | > | 34.190 | | 1.143 | | 380 | | 43 | 88 | 37 | 77 | 45 | | 87 | 48 | | 53 |  |
| - | *direct repeat 2* | 34.343 | > | 34.587 | | 486 | | - | | - | - | - | - | - | | - | - | | - |  |
| 40 | *MolaGV-ORF-40* | 34.584 | < | 34.808 | | 225 | | 74 | | 44 | 63 | 37b^+^ | 32 | 46 | | 61 | - | | - |  |
| 41 | *cp50-like* | 34.837 | > | 38.034 | | 3.198 | | 1065 | | 45 | 54 | 38 | 44 | 48 | | 36 | 50 | | 29 |  |
| 42 | *cp52-like* | 38.041 | < | 38.826 | | 786 | | 261 | | 47 | 77 | 39 | 77 | 50 | | 85 | 52 | | 63 |  |
| 43 | *pif-7* | 38.871 | > | 39.041 | | 171 | | 56 | | 48 | 79 | 40 | 65 | 51 | | 82 | 53 | | 43 |  |
| 44 | *v-ubq* | 39.042 | < | 39.284 | | 243 | | 80 | | 50 | 95 | 41 | 96 | 52 | | 97 | 54 | | 83 |  |
| 45 | *odv-ec43* | 39.374 | > | 40.435 | | 1.062 | | 353 | | 51 | 76 | 42 | 69 | 53 | | 73 | 55 | | 40 |  |
| 46 | *MolaGV-ORF-46* | 40.401 | > | 40.772 | | 372 | | 123 | | 52 | 82 | 43 | 72 | 54 | | 82 | - | | - |  |
| 47 | *39k* | 40.831 | < | 41.694 | | 864 | | 287 | | 53 | 77 | 44 | 69 | 55 | | 77 | 57 | | 21 |  |
| 48 | *lef-11* | 41.663 | < | 41.962 | | 300 | | 99 | | 54 | 84 | 45 | 74 | 56 | | 81 | 58 | | 49 |  |
| 49 | *MolaGV-ORF-49* | 42.444 | > | 43.265 | | 822 | | 273 | | 55 | 39 | 47 | 29 | 57 | | 41 | - | | - |  |
| 50 | *MolaGV-ORF-50* | 43.332 | < | 43.847 | | 516 | | 171 | | 60 | 70 | - | - | 64 | | 65 | - | | - |  |
| 51 | *sod* | 44.008 | < | 44.481 | | 474 | | 157 | | 64 | 82 | 50 | 68 | 68 | | 81 | 59 | | 62 |  |
| 52 | *MolaGV-ORF-52* | 44.516 | < | 44.668 | | 153 | | 50 | | 67 | 60 | - | - | 70 | | 62 | - | | - |  |
| 53 | *MolaGV-ORF-53* | 44.712 | > | 45.239 | | 528 | | 175 | | 70 | 57 | - | - | 71 | | 59 | - | | - |  |
| 54 | *MolaGV-ORF-54* | 45.271 | > | 46.299 | | 1.029 | | 342 | | 71 | 79 | - | - | 72 | | 78 | - | | - |  |
| 55 | *MolaGV-ORF-55* | 46.375 | > | 47.763 | | 1.389 | | 462 | | 72 | 65 | 56 | 46 | 73 | | 63 | - | | - |  |
| 56 | *cp65-like* | 47.824 | > | 48.018 | | 195 | | 64 | | 74 | 63 | 58 | 55 | 75 | | 66 | 65 | | 41 |  |
| - | *direct repeat 3* | 48.188 | > | 48.653 | | 884 | | - | | - | - | - | - | - | | - | - | | - |  |
| 57 | *MolaGV-ORF-57* | 48.939 | > | 50.078 | | 1.140 | | 379 | | 164 | 36 | 128 | 17 | 61 | | 23 | - | | - |  |
| 58 | *bro-a* | 51.247 | > | 52.005 | | 759 | | 252 | | 76 | 66 | 60 | 46 | 76 | | 68 | - | | - |  |
| 59 | *p74* | 52.034 | > | 54.184 | | 2.151 | | 716 | | 77 | 78 | 61 | 66 | 77 | | 80 | 60 | | 42 |  |
| 60 | *cp62-like* | 54.181 | < | 54.480 | | 300 | | 99 | | 78 | 82 | 62 | 73 | 77b^+^ | | 60 | 62 | | 50 |  |
| 61 | *p47* | 54.561 | > | 55.754 | | 1.194 | | 397 | | 79 | 79 | 63 | 74 | 78 | | 82 | 68 | | 54 |  |
| 62* | *DNA polymerase III delta prime subunit* | 55.772 | < | 56.065 | | 294 | | 97 | | - | - | - | - | - | | - | - | | - |  |
| 63 | *nudix* | 55.941 | > | 56.612 | | 672 | | 223 | | 82 | 95 | 64 | 91 | 79 | | 95 | 69 | | 65 |  |
| 64 | *p24* | 56.605 | > | 57.174 | | 570 | | 189 | | 83 | 79 | 65 | 66 | 80 | | 81 | 71 | | 51 |  |
| 65 | *38.7kDa* | 57.185 | < | 57.736 | | 552 | | 183 | | 84 | 55 | 66 | 63 | 81 | | 54 | 73 | | 26 |  |
| 66 | *lef-1* | 57.744 | < | 58.457 | | 714 | | 237 | | 85 | 84 | 67 | 70 | 82 | | 82 | 74 | | 62 |  |
| 67 | *MolaGV-ORF-67* | 58.522 | > | 59.127 | | 606 | | 201 | | 86 | 78 | 68 | 50 | 83 | | 69 | - | | - |  |
| 68 | *pif-1* | 59.145 | > | 60.770 | | 1.626 | | 541 | | 87 | 79 | 69 | 59 | 84 | | 79 | 75 | | 47 |  |
| 69 | *fgf-1* | 60.802 | < | 61.503 | | 702 | | 233 | | 88 | 53 | 70 | 42 | 85 | | 55 | 76 | | 24 |  |
| 70 | *MolaGV-ORF-70* | 61.550 | < | 61.891 | | 342 | | 113 | | 89 | 59 | - | - | 86 | | 54 | - | | - |  |
| 71 | *MolaGV-ORF-71* | 62.002 | > | 62.499 | | 498 | | 165 | | 90 | 66 | 72 | 38 | 87 | | 64 | 79 | | 32 |  |
| 72 | *lef-6* | 62.509 | < | 62.835 | | 327 | | 108 | | 91 | 76 | 73 | 66 | 88 | | 74 | 80 | | 38 |  |
| 73 | *dbp-1* | 62.867 | < | 63.706 | | 840 | | 279 | | 92 | 69 | 74 | 37 | 89 | | 65 | 81 | | 19 |  |
| 74 | *MolaGV-ORF-74* | 63.797 | < | 64.009 | | 213 | | 70 | | 93 | 79 | - | - | 89b^+^ | | 78 | - | | - |  |
| 75 | *MolaGV-ORF-75* | 63.970 | < | 64.710 | | 741 | | 246 | | 94 | 59 | 76 | 41 | 90 | | 57 | - | | - |  |
| 76 | *p48* | 64.709 | > | 65.827 | | 1.119 | | 372 | | 95 | 88 | 75 | 80 | 91 | | 89 | 83 | | 55 |  |
| 77 | *p12* | 65.841 | > | 66.194 | | 354 | | 117 | | 96 | 70 | 77 | 59 | 92 | | 71 | 84 | | 41 |  |
| 78 | *p40* | 66.246 | > | 67.355 | | 1.110 | | 369 | | 97 | 86 | 78 | 76 | 93 | | 84 | 85 | | 50 |  |
| 79 | *p6.9* | 67.395 | > | 67.577 | | 183 | | 60 | | 98 | 93 | 79 | 85 | 94 | | 93 | 86 | | 55 |  |
| 80 | *lef-5* | 67.628 | < | 68.467 | | 840 | | 279 | | 99 | 76 | 80 | 70 | 95 | | 80 | 87 | | 49 |  |
| 81 | *38k* | 68.390 | > | 69.316 | | 927 | | 308 | | 100 | 72 | 81 | 64 | 96 | | 75 | 88 | | 48 |  |
| 82 | *pif-4* | 69.335 | < | 69.808 | | 474 | | 157 | | 101 | 87 | 82 | 77 | 97 | | 86 | 89 | | 49 |  |
| 83 | *dna-helicase* | 69.807 | > | 73.295 | | 3.489 | | 1162 | | 102 | 84 | 83 | 75 | 98 | | 82 | 90 | | 38 |  |
| 84 | *odv-e25* | 73.353 | < | 74.015 | | 663 | | 220 | | 103 | 89 | 85 | 79 | 99 | | 89 | 91 | | 65 |  |
| 85 | *p18* | 74.056 | < | 74.532 | | 477 | | 158 | | 104 | 80 | 86 | 62 | 100 | | 79 | 92 | | 35 |  |
| 86 | *p33* | 74.650 | > | 75.405 | | 756 | | 251 | | 105 | 90 | 87 | 71 | 101 | | 90 | 93 | | 53 |  |
| 87 | *chaB* | 75.425 | < | 75.682 | | 258 | | 85 | | 106 | 96 | 88 | 85 | 102 | | 96 | - | | - |  |
| 88 | *MolaGV-ORF-88* | 75.715 | < | 75.936 | | 222 | | 73 | | 107 | 56 | - | - | - | | - | - | | - |  |
| 89 | *MolaGV-ORF-89* | 76.136 | > | 76.486 | | 351 | | 116 | | 109 | 60 | - | - | 106 | | 65 | - | | - |  |
| 90 | *lef-4* | 76.533 | < | 77.876 | | 1.344 | | 447 | | 114 | 73 | 91 | 64 | 110 | | 74 | 95 | | 43 |  |
| 91 | *vp39* | 77.928 | > | 78.893 | | 966 | | 321 | | 115 | 80 | 92 | 73 | 111 | | 80 | 96 | | 39 |  |
| 92 | *odv-ec27* | 79.001 | > | 79.870 | | 870 | | 289 | | 116 | 89 | 93 | 80 | 112 | | 88 | 97 | | 45 |  |
| - | *direct repeat 4* | 79.984 | > | 80.140 | | 314 | | - | | - | - | - | - | - | | - | - | | - |  |
| 95 | *cp99-like* | 80.277 | < | 81.404 | | 1.128 | | 375 | | 117 | 68 | 94 | 50 | 113 | | 66 | 99 | | 27 |  |
| 94* | *MolaGV-ORF-94* | 81.473 | > | 81.655 | | 183 | | 60 | | - | - | - | - | - | | - | - | | - |  |
| 95 | *bro-b* | 81.726 | < | 82.886 | | 1.161 | | 386 | | 118 | 82 | 95 | 62 | 114 | | 80 | - | | - |  |
| 96 | *MolaGV-ORF-96* | 83.021 | > | 84.130 | | 1.110 | | 369 | | 119 | 64 | 96 | 49 | 115 | | 64 | - | | - |  |
| 97 | *cp100-like* | 84.251 | > | 84.631 | | 381 | | 126 | | 120 | 76 | 97 | 60 | 116 | | 77 | 100 | | 31 |  |
| 98 | *MolaGV-ORF-98* | 84.677 | < | 85.132 | | 456 | | 151 | | 121 | 66 | - | - | 117 | | 55 | - | | - |  |
| 99 | *vp91* | 85.258 | < | 87.468 | | 2.211 | | 736 | | 122 | 70 | 100 | 57 | 118 | | 70 | 101 | | 42 |  |
| 100 | *cp102-like* | 87.434 | > | 87.892 | | 459 | | 152 | | 123 | 68 | 101 | 54 | 119 | | 70 | 102 | | 41 |  |
| 101 | *ac81-like* | 87.912 | > | 88.475 | | 564 | | 187 | | 124 | 91 | 102 | 79 | 120 | | 89 | 103 | | 58 |  |
| 102 | *gp41* | 88.532 | > | 89.407 | | 876 | | 291 | | 125 | 81 | 103 | 73 | 121 | | 82 | 104 | | 50 |  |
| 103 | *ac78-like* | 89.474 | > | 89.797 | | 324 | | 107 | | 126 | 69 | 104 | 58 | 122 | | 70 | 105 | | 22 |  |
| 104 | *vlf-1* | 89.790 | > | 90.908 | | 1.119 | | 372 | | 127 | 83 | 105 | 80 | 123 | | 82 | 106 | | 54 |  |
| 105 | *MolaGV-ORF-105* | 90.912 | < | 91.454 | | 543 | | 180 | | 128 | 71 | 107 | 59 | 124 | | 72 | - | | - |  |
| 106 | *cp107-like* | 91.495 | > | 91.752 | | 258 | | 85 | | 129 | 94 | 106 | 91 | 125 | | 93 | 107 | | 61 |  |
| 107 | *cp108-like* | 91.829 | > | 92.266 | | 438 | | 145 | | 130 | 77 | 108 | 68 | 126 | | 78 | 108 | | 36 |  |
| 108 | *iap-3* | 92.322 | < | 93.053 | | 732 | | 243 | | 143 | 24 | 114 | 27 | 137 | | 26 | 17 | | 39 |  |
| 109 | *dna-pol* | 93.148 | < | 96.381 | | 3.234 | | 1077 | | 138 | 83 | 109 | 75 | 132 | | 83 | 111 | | 54 |  |
| 110 | *desmoplakin* | 96.380 | > | 98.314 | | 1.935 | | 644 | | 139 | 62 | 110 | 47 | 133 | | 57 | 112 | | 51 |  |
| 111 | *lef-3* | 98.445 | < | 99.500 | | 1.056 | | 351 | | 140 | 56 | 111 | 48 | 134 | | 54 | 113 | | 25 |  |
| 112 | *pif-6* | 99.469 | > | 99.879 | | 411 | | 136 | | 141 | 79 | 112 | 61 | 135 | | 77 | 114 | | 44 |  |
| 113 | *MolaGV-ORF-113* | 99.927 | > | 100.451 | | 525 | | 174 | | 142 | 53 | 113 | 38 | 136 | | 50 | - | | - |  |
| 114 | *iap-5* | 100.515 | > | 101.366 | | 852 | | 283 | | - | - | - | - | - | | - | 116 | | 33 |  |
| 115 | *lef-9* | 101.440 | > | 102.915 | | 1.476 | | 491 | | 145 | 90 | 115 | 80 | 139 | | 90 | 117 | | 63 |  |
| 116 | *fp25k* | 102.962 | > | 103.393 | | 432 | | 143 | | 146 | 86 | 116 | 80 | 140 | | 83 | 118 | | 48 |  |
| 117 | *dna-ligase* | 103.395 | < | 104.981 | | 1.587 | | 528 | | 148 | 83 | 117 | 68 | 141 | | 81 | 120 | | 42 |  |
| 118 | *cp121-like* | 105.160 | > | 105.414 | | 255 | | 84 | | 149 | 61 | 118 | 69 | 142 | | 60 | 121 | | 36 |  |
| 119 | *MolaGV-ORF-119* | 105.481 | < | 105.681 | | 201 | | 66 | | 150 | 86 | 119 | 61 | 143 | | 86 | - | | - |  |
| 120 | *fgf-2* | 105.740 | < | 106.984 | | 1.245 | | 414 | | 151 | 66 | 120 | 51 | 144 | | 62 | 123 | | 26 |  |
| 121 | *alk-exo* | 107.115 | > | 108.353 | | 1.239 | | 412 | | 152 | 73 | 121 | 60 | 145 | | 70 | 125 | | 40 |  |
| 122 | *hel-2* | 108.396 | > | 109.757 | | 1.362 | | 453 | | 153 | 75 | 122 | 68 | 146 | | 74 | 126 | | 47 |  |
| 123 | *MolaGV-ORF-123* | 109.834 | > | 110.850 | | 1.017 | | 338 | | 154 | 62 | 123 | 50 | 147 | | 63 | - | | - |  |
| 124 | *lef-8* | 110.847 | < | 113.441 | | 2.595 | | 864 | | 155 | 86 | 124 | 80 | 148 | | 87 | 131 | | 61 |  |
| 125 | *odv-e66* | 113.496 | < | 115.496 | | 2.001 | | 666 | | 156 | 78 | 126 | 65 | 149 | | 78 | 37 | | 40 |  |
| - | *hr1* | 115.546 | > | 115.964 | | 836 | | - | | - | - | - | - | - | | - | - | | - |  |
| 126 | *enhancin-1* | 116.014 | < | 118.497 | | 2.484 | | 827 | | 157 | 63 | - | - | 150 | | 63 | - | | - |  |
| 127 | *enhancin-3* | 118.540 | > | 121.206 | | 2.667 | | 888 | | 159 | 69 | - | - | 154 | | 71 | - | | - |  |
| 128 | *MolaGV-ORF-128* | 121.295 | < | 121.513 | | 219 | | 72 | | 163 | 76 | - | - | 160 | | 72 | - | | - |  |
| 129 | *MolaGV-ORF-129* | 121.706 | > | 123.169 | | 1.464 | | 487 | | 164 | 70 | 128 | 55 | 161 | | 69 | - | | - |  |
| 130 | *MolaGV-ORF-130* | 123.092 | < | 123.766 | | 675 | | 224 | | 165 | 49 | 130 | 35 | 162 | | 49 | - | | - |  |
| - | *direct repeat 5* | 124.012 | > | 124.059 | | 96 | | 31 | | - | - | - | - | - | | - | - | | - |  |
| 131 | *MolaGV-ORF-131* | 124.306 | > | 124.653 | | 348 | | 115 | | 169 | 76 | 131 | 66 | 165 | | 75 | - | | - |  |
| 132 | *enhancin-4* | 124.685 | < | 127.258 | | 2.574 | | 857 | | 170 | 68 | 132 | 40 | 166 | | 66 | - | | - |  |
| - | *hr2* | 127.322 | > | 127.589 | | 535 | | - | | - | - | - | - | - | | - | - | | - |  |
| 133 | *MolaGV-ORF-133* | 127.629 | < | 127.829 | | 201 | | 66 | | 172 | 62 | - | - | 170 | | 60 | - | | - |  |
| 134 | *ac53-like* | 127.816 | > | 128.232 | | 417 | | 138 | | 173 | 72 | 135 | 57 | 171 | | 71 | 134 | | 38 |  |
| 135 | *cp135-like* | 128.234 | < | 129.361 | | 1.128 | | 375 | | 174 | 65 | 136 | 58 | 172 | | 60 | 135 | | 28 |  |
| 136 | *MolaGV-ORF-136* | 129.365 | < | 129.568 | | 204 | | 67 | | 175 | 78 | 137 | 63 | 173 | | 73 | - | | - |  |
| 137 | *lef-10* | 129.546 | > | 129.758 | | 213 | | 70 | | 176 | 89 | 138 | 69 | 174 | | 91 | 137 | | 27 |  |
| 138 | *vp1054* | 129.637 | > | 130.614 | | 978 | | 325 | | 177 | 87 | 139 | 76 | 175 | | 84 | 138 | | 45 |  |
| 139 | *MolaGV-ORF-139* | 130.696 | > | 130.878 | | 183 | | 60 | | 178 | 85 | 140 | 66 | 176 | | 78 | - | | - |  |
| 140* | *MolaGV-ORF-140* | 130.806 | < | 130.967 | | 162 | | 53 | | - | - | - | - | - | | - | - | | - |  |
| 141 | *MolaGV-ORF-141* | 130.980 | > | 131.282 | | 303 | | 100 | | 179 | 49 | 141 | 33 | 177 | | 51 | - | | - |  |
| 142 | *fgf-3* | 131.323 | > | 132.270 | | 948 | | 315 | | 180 | 76 | 142 | 51 | 178 | | 74 | 140 | | 20 |  |
| - | *direct repeat 6* | 131.414 | > | 131.468 | | 110 | | - | | - | - | - | - | - | | - | - | | - |  |
| 143 | *MolaGV-ORF-143* | 132.372 | > | 132.983 | | 612 | | 203 | | 181 | 62 | 143b | 31 | 179 | | 60 | - | | - |  |
| - | *direct repeat 7* | 132.582 | > | 132.603 | | 22 | | - | | - | - | - | - | - | | - | - | | - |  |
| 144 | *me53* | 133.027 | > | 133.923 | | 897 | | 298 | | 182 | 75 | 144 | 64 | 180 | | 75 | 143 | | 35 |  |
| 145 | *MolaGV-ORF-145* | 133.925 | > | 134.254 | | 330 | | 109 | | 183 | 79 | 145 | 63 | 181 | | 76 | - | | - |  |

*: unique gene

^+^: not annotated in the Genbank database genome
