# Supplementary material for: A Novel Betabaculovirus Isolated from the Monocot Pest Mocis latipes (Lepidoptera: Noctuidae) and the Evolution of Multiple-Copy Genes
Source: Viruses. 2018 Mar 16;10(3):134. doi: 10.3390/v10030134 (PMC5869527; doi:10.3390/v10030134)
Supplement: Supplementary file 1 [file viruses-10-00134-s001.zip › Table S3.docx]

|  | *lef-8* | *lef-9* | *granulin* |
| --- | --- | --- | --- |
| PsunGV | 0,274 | 0,222 | 0,187 |
| TnGV | 0,277 | 0,222 | 0,187 |
| HearGV | 0,309 | 0,214 | 0,189 |
| XecnGV | 0,315 | 0,221 | 0,195 |
| SpfrGV | 0,407 | 0,302 | 0,206 |
| MyunGV | 0,453 | 0,315 | 0,201 |

Table S3: Evolutionary Divergence between MolaGV-related baculovirus using Kimura 2-parameter model of evolution.

.
