# Supplementary material for: A Novel Betabaculovirus Isolated from the Monocot Pest Mocis latipes (Lepidoptera: Noctuidae) and the Evolution of Multiple-Copy Genes
Source: Viruses. 2018 Mar 16;10(3):134. doi: 10.3390/v10030134 (PMC5869527; doi:10.3390/v10030134)
Supplement: Supplementary file 1 [file viruses-10-00134-s001.zip › Table S4.docx]

**Table S4**. Distribution of the chitinase and cathepsin in betabaculovirus genomes.

| **Clade** | **Betabaculovirus** | **Acronym** | **ChiA** | **V-Cath** |
| --- | --- | --- | --- | --- |
| A | Agrotis segetum granulovirus isolate L1 | AgseGV-L1 | + | + |
|  | Plutella xylostella granulovirus | PlxyGV | - | - |
|  | Spodoptera litura granulovirus K1 | SpliGV | - | - |
|  | Mythimna unipuncta granulovirus | MyunGV | - | - |
|  | Spodoptera frugiperda granulovirus | SpfrGV | - | - |
|  | Mocis latipes granulovirus | MolaGV | - | - |
|  | Pseudaletia unipuncta granulovirus | PsunGV | + | + |
|  | Trichoplusia ni granulovirus | TnGV | + | + |
|  | Helicoverpa armigera granulovirus | HaGV | - | + |
|  | Xestia c-nigrum granulovirus | XcGV | + | + |
| B | Epinotia aporema granulovirus | EpapGV | + | + |
|  | Plodia interpunctella granulovirus | PiGV | - | + |
|  | Clostera anastomosis granulovirus | ClasGV | + | + |
|  | Clostera anachoreta granulovirus | ClanGV | + | + |
|  | Cydia pomonella granulovirus | CpGV | + | + |
|  | Cryptophlebia leucotreta granulovirus CV3 | CrleGV | + | + |
|  | Cnaphalocrocis medinalis granulovirus | CnmeGV | - | - |
|  | Adoxophyes orana granulovirus | AdorGV | - | - |
|  | Phthorimaea operculella granulovirus | PhopGV | - | - |
|  | Diatraea saccharalis granulovirus | DisaGV | - | - |
|  | Pieris rapae granulovirus isolate E3 | PiraGV-E3 | + | + |
|  | Choristoneura occidentalis granulovirus | ChocGV | - | - |
|  | Clostera anastomosis granulovirus B | ClasGV-B | - | - |
|  | Erinnyis ello granulovirus | ErelGV | - | - |
