# Supplementary figures and images for: A Novel Betabaculovirus Isolated from the Monocot Pest Mocis latipes (Lepidoptera: Noctuidae) and the Evolution of Multiple-Copy Genes
Source: Viruses. 2018 Mar 16;10(3):134. doi: 10.3390/v10030134 (PMC5869527; doi:10.3390/v10030134)

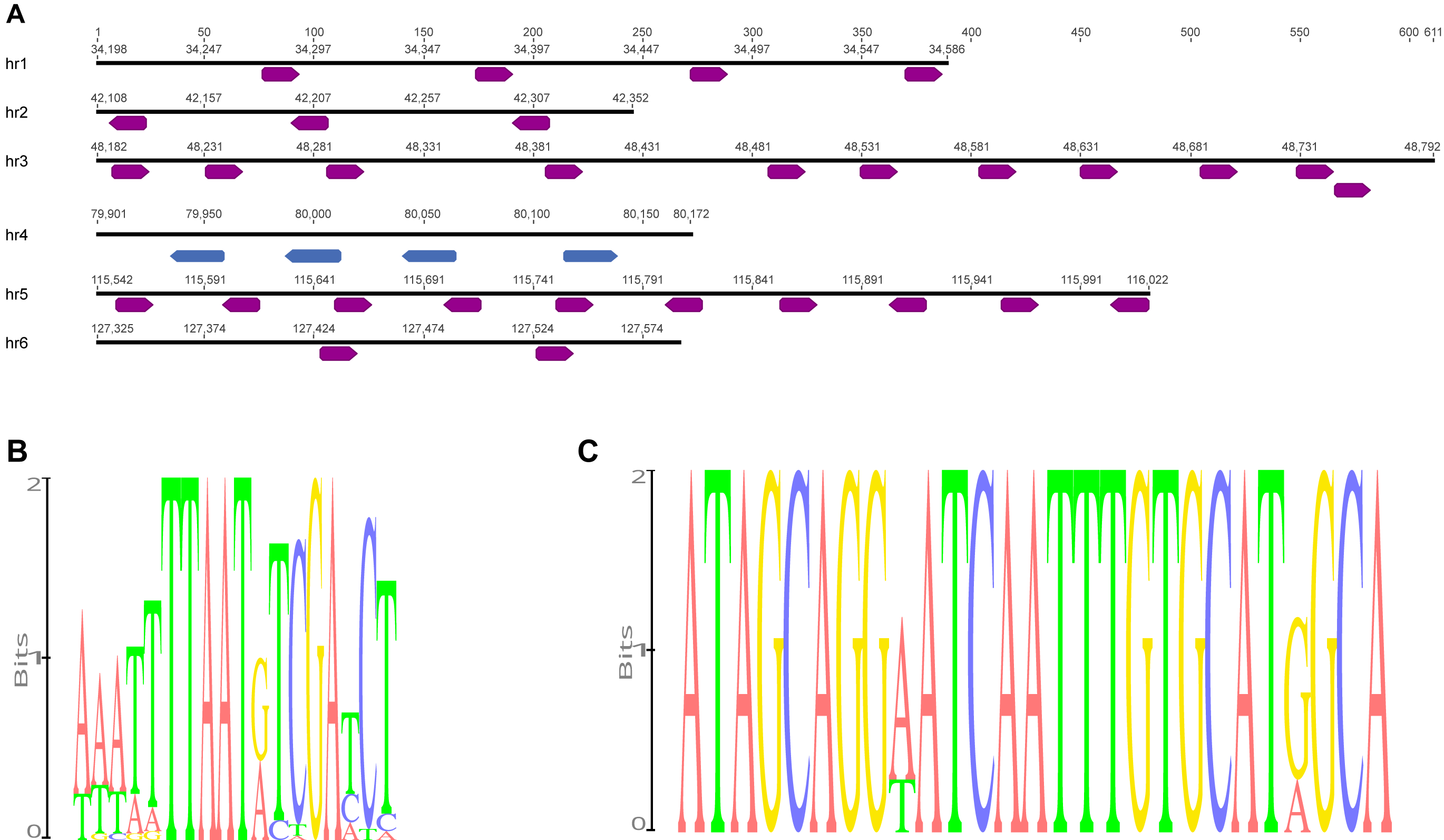

Supplement: Supplementary file 1 [file viruses-10-00134-s001.zip › Figure S1.tif]
